# Co-Motif-Engineered RuO_2_ Nanosheets for Robust and Efficient Acidic Oxygen Evolution

**DOI:** 10.1021/acsami.5c00773

**Published:** 2025-03-19

**Authors:** Jiandong Hu, Le Tong, Yanlin Jia, Ziye Li, Haowei Yang, Yang Wang, Wenhui Luo, Yejun Li, Yong Pang, Shiyun Xiong, Zhi Liang Zhao, Qi Wang

**Affiliations:** ‡ School of Materials Science and Engineering, 506598 Central South University, Changsha, Hunan 410083, People’s Republic of China; § National Energy Key Laboratory for New Hydrogen-Ammonia Energy Technologies, Foshan Xianhu Laboratory, Foshan, Guangdong 528200, People’s Republic of China; ∥ School of Materials and Energy, 530072 Guangdong University of Technology, Guangzhou, Guangdong 510006, People’s Republic of China; ⊥ Department of Materials Science and Engineering, 53025 City University of Hong Kong, Kowloon 999077, Hong Kong Special Administrative Region of the People’s Republic of China

**Keywords:** electrocatalyst, Co-doped RuO_2_ nanosheets, acidic oxygen evolution reaction, oxygen vacancy engineering, AEM pathway

## Abstract

The development of efficient and reliable acidic oxygen evolution reaction (OER) electrocatalysts represents a crucial step in the process of water electrolysis. RuO_2_, a benchmark OER catalyst, suffers from limited large-scale applicability due to its tendency toward the less stable lattice oxygen mechanism (LOM). This work reports the synthesis of Co-doped RuO_2_ nanosheets with a unique porous morphology composed of interconnected grains via a facile molten salt method. Co doping modulates the grain size, effectively increasing the specific surface area and introducing oxygen vacancies. These oxygen vacancies, coupled with the Co dopants, form Co–O­(V) motifs that tune the electronic configuration of Ru. This structural engineering promotes a shift in the OER mechanism from the detrimental LOM pathway to the more efficient adsorbate evolution mechanism (AEM), significantly enhancing the stability of the RuO_2_ matrix in acidic environments. The optimized Co_0.108_–RuO_2_ catalyst exhibits a low overpotential of 214 mV at 10 mA cm^–2^ and remarkable stability over commercial RuO_2_ and undoped counterparts, owing to the synergistic effect of the increased surface area, Co–O­(V) motifs, and favored AEM pathway. This strategy of utilizing Co doping to engineer morphology, electronic structure, and reaction mechanism offers a promising avenue for developing high-performance OER electrocatalysts.

## Graphical abstract

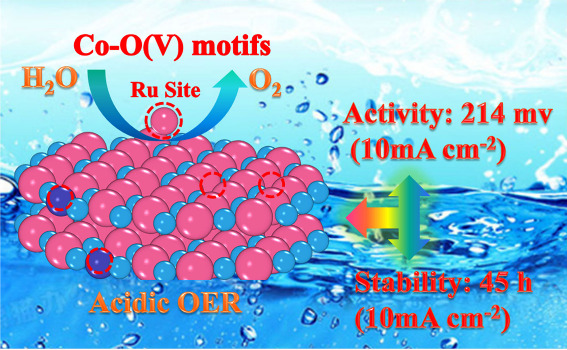

## 1 Introduction

Proton-exchange membrane water electrolyzers (PEMWEs) offer advantages over traditional alkaline electrolyzers due to their higher current densities, faster response times, and compact design.^1–4^ However, the development of cost-effective, highly active, and stable acidic oxygen evolution reaction (OER) electrocatalysts remains a significant challenge for large-scale hydrogen production using PEMWEs.^5–8^ Iridium dioxide (IrO_2_) catalysts demonstrate moderate stability when employed in commercial PEMWEs, their sub-optimal activity necessitates the use of high anodic catalyst loading, which in turn increases the cost of PEMWEs and presents a challenge to large-scale commercial application.^9–11^ While RuO_2_ is a promising alternative to IrO_2_ due to its lower cost and high intrinsic activity,^12–15^ its instability hinders its widespread industrial application. It is widely accepted that the mechanism underlying the degradation of its stability is the highly covalent triggering of the lattice oxygen oxidation mechanism (LOM) by the Ru–O bond. This process leads to the formation of oxygen vacancies (O_V_) and the leaching of reactive ruthenium substances (soluble RuO_4_ substances), which ultimately accelerate the collapse of the crystal structure.^16–20^ Consequently, the design of high-performance and highly stable Ru-based oxide catalysts represents a significant challenge.

Engineering the morphology of nanomaterials can significantly impact their catalytic performance. Two-dimensional (2D) nanosheets offer numerous electrochemically active sites and enhanced surface functionality due to the high proportion of surface atoms. This leads to increased surface area, improved mechanical flexibility, faster interfacial charge transfer, and more facile electrochemical reactions.^21–24^ Besides, defect engineering, particularly the introduction of oxygen vacancies, is a proven strategy for enhancing catalytic activity.^25^ Oxygen vacancies provide abundant active sites with high intrinsic activity and modulate the local electronic structure, optimizing adsorption and activation energies and reducing covalency.^26–28^ However, precisely controlling vacancy concentration remains a challenge. Doping with heteroatoms in different chemical valence is a powerful tool for tailoring both morphology and generating oxygen vacancy, thereby modifying the catalyst’s reactivity.^5,29–31^ For instance, interstitial Si doping has been shown to stabilize Ru sites in RuO_2_ and suppress LOM.^1^ Similarly, W and Er co-doping can modulate the electronic structure and oxygen vacancy formation energy, leading to improved OER performance.^32^ However, there remains a need for further exploration of doping strategies to precisely control both morphology and oxygen vacancy concentration for optimal OER activity and stability.

In this study, Co-doped RuO_2_ ultrathin 2D nanosheets with oxygen vacancies were synthesized using a facile and rapid molten salt method. This synthesis yields a porous nanosheet structure composed of interconnected grains, where the grain size is modulated by Co doping, effectively increasing the specific surface area and the proportion of defects. The controlled introduction of Co not only influences the morphology but also introduces oxygen vacancies, forming Co–O­(V) motifs that adjust the electronic configuration of Ru. The resulting decrease in the Ru/O coordination ratio favors the reduction of Ru from higher oxidation states (Ru > 4+) and prevents the overoxidation of RuO_2_ to RuO_4_, thus suppressing the LOM pathway and promoting the adsorbate evolution mechanism (AEM) pathway. Density functional theory (DFT) calculations reveal that the optimal concentration of oxygen vacancies and Co doping within the RuO_2_ lattice facilitates the AEM pathway, modulates the electronic structure, and prevents overoxidation of Ru species. Thus, these Co-doped RuO_2_ nanosheets with oxygen vacancies offer a promising approach for developing efficient and stable Ru-based OER electrocatalysts.

## 2 Materials and Methods

### 2.1 Materials

Anhydrous ruthenium chloride (RuCl_3_, 37%, Adamas, AR), cobalt nitrate hexahydrate [Co­(NO_3_)_2_·6H_2_O, AR], and Nafion (5 wt %) were procured from Shanghai Titan Technology. Sulfuric acid (H_2_SO_4_, 98%), anhydrous ethanol, and sodium nitrate (NaNO_3_, 99.0%, AR) were procured from Sinopharm Chemical Reagent. Carbon paper was supplied by Shanghai Huayu Instrumentation. All reagents were of analytical grade and did not require further purification.

### 2.2 Synthesis of Ruthenium Dioxide Nanosheets (RuO_2_)

Fifteen ml of alcohol solution was prepared with RuCl_3_ in a specific ratio (the concentration of Ru in the solution was 92.5 mg/mL) and set aside. Then, 400 mL of RuCl_3_ solution was added to an 8 mL glass vial for sonication. Five g NaNO_3_ (AR) was added to a 25 mL beaker, and the solution in the glass vial was transferred to the surface of NaNO_3_ (AR) and then dried in an oven at 60 °C for 30 min. The dried reagent mixture was transferred to a muffle furnace at 350 °C and held at this temperature for 35 min before being removed. After cooling to room temperature, the product was collected by sonication and centrifuged into 5 mL centrifuge tubes. The centrifuge tubes containing the samples are then dried in a vacuum oven at 60 °C for 12 h.

### 2.3 Synthesis of Cobalt-Doped Ruthenium Dioxide Nanosheets (Co_*x*_–RuO_2_)

RuCl_3_ was prepared by adding 15 mL of alcohol to RuCl_3_ to form a certain proportion of the solution and set aside. Then, a certain amount of RuCl_3_ solution (385 μL) and Co­(NO_3_)_2_.6H_2_O (AR) were mixed in specific proportions (the molar ratios of Ru/Co is 1:0.033, 1:0.047, 1:0.083, 1:0.108, and 1:0.167, respectively) in an 8 mL glass vial and sonicated to achieve uniform mixing. Next, 5 g of NaNO_3_ (AR) was transferred to a 25 mL beaker, and the solution in the above glass vial was transferred to the surface of NaNO_3_ (AR) and then dried in a desiccator at 60 °C for 30 min. The dried reagent mixture was transferred to a muffle furnace at 350 °C for 35 min, then removed and cooled at room temperature. The product was collected by sonication and centrifugation into 5 mL centrifuge tubes, and the centrifuge tubes containing the samples were placed in a vacuum drying oven at 60 °C for 12 h.

### 2.4 Characterization

X-ray diffraction (XRD) patterns were obtained using a Panaco Empyrean with a Cu Kα radiation source and a scanning range of 5–80°. Inductively coupled plasma mass spectrometry (ICP–MS) was tested using a U.S. Aglient 5110 (OES) unit. The samples were subjected to conformal and nanoscale structural characterization through the use of focused ion beam scanning electron microscopy (SEM, TESCAN-AMBER) and JEOL ARM 300 double Cs-corrected transmission electron microscopy (TEM) operated 300 kV. Atomic force microscopy (AFM) was employed to obtain morphological measurements, utilizing a Bruker edge AFM. A Brunauer–Emmett–Teller (BET) specific surface area and pore size analysis was conducted using a Micromeritics ASAP 2460. The surface composition and valence characterization of the samples was conducted using X-ray photoelectron spectroscopy (XPS) with the Shimadzu AXIS SUPRA+ model. The presence of oxygen vacancies was identified through the use of paramagnetic resonance spectroscopy (EPR) on a Bruker EMXPLUS instrument.

### 2.5 Electrochemical Measurements

A mixture of 2 mg catalyst, 10 μL Nafion (5 wt %), and 700 μL anhydrous ethanol was configured and the solution was thoroughly mixed using a sonicator for 30 min. Thereafter, 100 μL of ink was transferred to the surface of a 1 cm^2^ area of carbon paper (catalyst loading ∼ 282 μg cm^–2^), dried using an infrared lamp, and set aside.

Electrochemical tests were performed on an electrochemical workstation (CHI 760E, Zhenhua, Shanghai, China) using a three-electrode system. The electrolyte was selected as 0.5 M H_2_SO_4_, the Hg/Hg_2_SO_4_ electrode was selected as a reference electrode, the Pt electrode (1 × 1 cm) was selected as a counter electrode, and the catalyst-coated carbon paper with a surface area of 1 cm^2^ was utilized as the working electrode. The catalyst loading on the carbon paper was 0.282 mg cm^–2^. The activation process was performed by cyclic voltammetry (CV) at a scan rate of 100 mV s^–1^, and the bilayer capacitance was determined by measuring the CV value from 20 to 120 mV s^–1^ (in continuous increments of 20 mV s^–1^. Linear scanning voltammetry (LSV) measurements were performed at a scan rate of 5 mV s^–1^, and all LSV curve measurements were performed with 95% *iR* compensation. Electrochemical impedance spectroscopy (EIS) was performed in the frequency range from 10 kHz to 0.1 Hz. The chronopotentiometric method, represented by the *V*–*t* curve, was selected for stability testing (at 10 mA cm^–2^).

The Faraday efficiency (FE) was measured by the drainage method as follows:

A constant current of 10 mA was applied to the H-type electrolytic cell and the test was started when the catalyst was stable. It was kept going for 2 h and the volume of oxygen collected was recorded every 20 min. The same procedure was repeated 4 times, and the average value was taken to calculate the final FE result.

The FE and theoretical oxygen production were determined using the following eqs 1 and 2, respectively:

1 $$\mathrm{FE}\left(O_{2},\%\right)=\frac{V_{O_{2}}\times n\times F}{V_{m}\times i\times t}\times 100\%$$

2 $$n\left(\mathrm{theoretical}O_{2},\mathrm{mol}\right)=\frac{i\times t}{n\times F}$$

where *V*_O_2__ is the volume of actual oxygen production (L), *n* is the number of electrons transferred during oxygen precipitation (*n* = 4), *F* is the Faraday’s constant (*F* = 96 485 C mol^–1^), *V*_m_ is the molar volume of the gas at room temperature (*V*_m_ = 22.4 L mol^–1^), *i* is the applied current (*i* = 0.01 A), and *t* is the time of the reaction (s).

### 2.6 Theoretical Calculations

The rutile structure of RuO_2_ exhibits the greatest stability and lowest energy at the RuO_2_ (110) surface, which was therefore selected as the model for the simulations. A (3 × 2) supercell with five atomic layers was constructed to model the structure, and a vacuum space of 15 Å was incorporated. The three uppermost layers were fully relaxed, while the two lowermost layers were maintained as bulk crystals. All calculations were performed using DFT from the Vienna *Ab Initio* Simulation Package (VASP), as well as describing electron-exchange-related interactions using Perdew–Burke–Ernzerhof (PBE) functions. In the course of the geometry optimization process, the energy cutoff for plane waves is 500 eV, and all structural relaxations are k-space integrated using a (1 × 1 × 1) *k*-point grid. The structural energies and force convergences involved are less than 1 × 10^–4^ eV and 0.02 eV/Å, respectively. As the models developed include magnetic atoms, it is necessary to consider the impact of spin polarization when calculating the energy of each model.

The Gibbs free energy for each step is defined as eq 3

3 $$\mathrm{Gibbs}=E_{\mathrm{DFT}}+E_{\mathrm{ZPE}}-TS$$

where *E*_DFT_, *E*_ZPE_, and *TS* denote the electron energy, zero point energy (ZPE), and entropy at room temperature (*T* = 298.15 K) calculated by DFT.

The two reaction mechanisms involved in the OER reaction are the AEM mechanism and the LOM mechanism, where the AEM mechanism reaction steps are eqs 4–7.

4 *+H2O=OH*+H++e−

5 OH*=O*+H++e−

6 O*+H2O=OOH*+H++e−

7 OOH*=*+O2+H++e−

The LOM mechanism reaction steps are eqs 8–10.

8 *+H2O=OH*+H++e−

9 O*=O*+H++e−

10 O*+OL=O2(g)+VO

## 3 Results and Discussion

The synthesis of Co-doped RuO_2_ nanosheets, denoted as Co_*x*_–RuO_2_ (where *x* represents the Co/Ru molar ratio), is depicted in Figure 1a (see section 2.3 for detailed procedures). The actual Co doping levels, as determined by ICP–MS, are presented in Table S1. These measured values are approximately consistent with the theoretical doping levels, with minor deviations attributed to the inherent challenge of preventing Co loss during the synthesis process. XRD analysis (Figure 1b and Figure S1) revealed a shift of diffraction peaks to higher angles with increasing Co doping up to a Co/Ru ratio of 0.167, revealed by EDS analysis. Beyond this ratio, phase separation occurs, with the formation of a Co_3_O_4_ phase (Figure S1). For Co doping levels below this threshold, the materials retain the rutile RuO_2_ crystal structure [Powder Diffraction File (PDF) 97-005-6007], as shown in Figure 1b. The peak shift to higher angles, highlighted in the inset of Figure 1b, confirms the successful incorporation of Co atoms into the Ru crystal lattice through substitutional doping, where Co atoms replace Ru atoms, causing lattice contraction.

**Figure 1:**
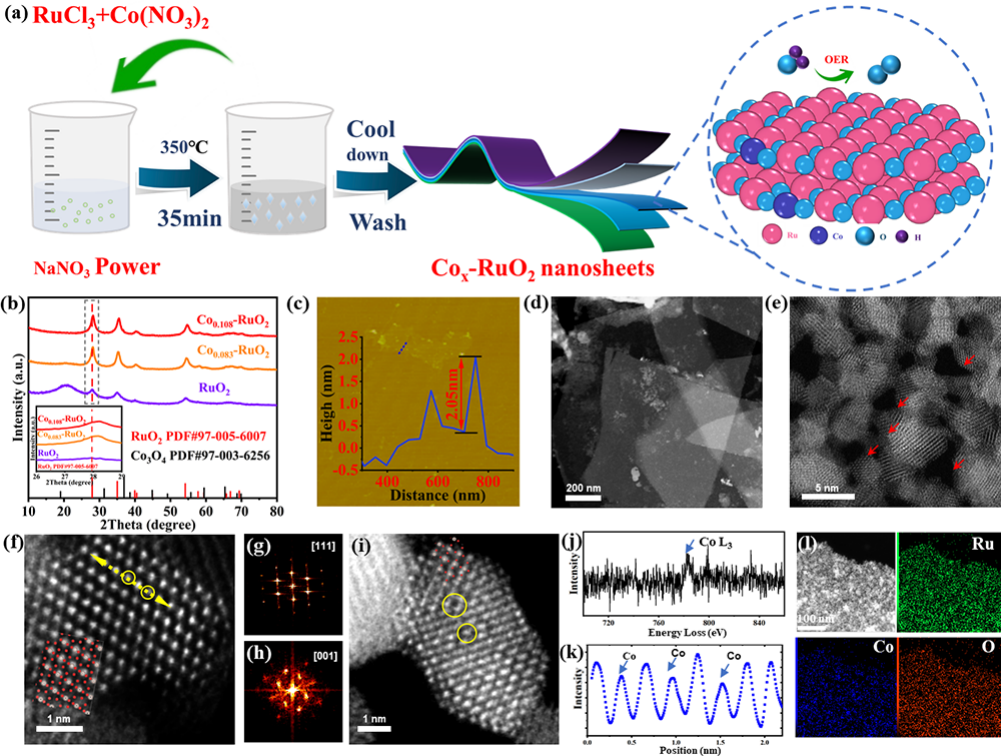
(a) Schematic representation of the synthesis of Co_*x*_–RuO_2_. (b) XRD spectra of RuO_2_, Co_0.083_–RuO_2_, and Co_0.108_–RuO_2_ (inset: magnified XRD spectra of the strongest peak of RuO_2_, Co_0.083_–RuO_2_, and Co_0.108_–RuO_2_, 26–29°). (c) AFM image of Co_0.108_–RuO_2_. (d) HR-STEM image of Co_0.108_–RuO_2_ (200 nm). (e) HR-STEM image of Co_0.108_–RuO_2_ (5 nm). (f) HR-STEM images of Co_0.108_–RuO_2_ (1 nm). (g) FFT of [111] zone axe in panel f. (h) FFT of [001] zone axe in panel f. (i) HR-STEM image of Co_0.108_–RuO_2_ (1 nm). (j) EELS at the Co L_3_-edge. (k) Corresponding line-scanning intensity profile of the yellow line in panel f. (l) EDS maps of Co_0.108_–RuO_2_ for Ru, Co, O, and the combined image.

Scanning transmission electron microscopy (STEM) and AFM were employed to characterize the morphology and atomic structure of the Co_0.108_–RuO_2_ catalyst. Figure 1d and Figures S3 and S4a confirm the nanosheet morphology, while AFM analysis (Figure 1c) reveals a thickness of approximately 2 nm. This ultrathin two-dimensional structure is expected to provide a larger electrochemically active surface area and more active sites compared to conventional nanomaterials, potentially enhancing reaction rates.^33,34^ High-magnification STEM images (panels a and b of Figure S4) show that the nanosheets are composed of interconnected particles, 1–5 nm in size. This arrangement creates abundant grain boundaries and a microporous structure (indicated by the red arrow in Figure 1e), and the corresponding pore size distribution results show that Co_0.108_–RuO_2_ nanosheets have a porous structure (Figure S6), facilitating mass transport, a crucial factor in catalytic reactions. Furthermore, analysis of the constituent grains revealed that these grains have (110) facets as the dominant exposed surfaces (Figure S4c). These structural features contribute to the high specific surface area of Co_0.108_–RuO_2_ (170.1 m^2^/g), determined by BET analysis (Figure S5), which significantly surpasses that of Co_0.083_–RuO_2_ (141.108 m^2^/g), commercial RuO_2_ (26.92 m^2^/g) and commercial IrO_2_ (14.19 m^2^/g), indicating a greater availability of active sites for the oxygen evolution reaction (OER).

High-resolution scanning transmission electron microscopy (HR-STEM) was used to investigate the atomic-scale location of Co dopants within the RuO_2_ structure. Figures 1f and 1i display the atomic arrangements of Co_0.108_–RuO_2_ along the [111] and [001] zone axes, respectively. The (110) crystal plane spacing in Co_0.108_–RuO_2_ is measured to be 3.157 Å (Figure 1f), smaller than the 3.168 Å spacing of pure rutile RuO_2_,^35^ consistent with the XRD results. Corresponding fast Fourier transforms (FFTs) are shown in panels g and h of Figure 1. These represent divergent electron diffraction patterns obtained using incident electron beams along the [111] and [001] band axes. Blurred lattice positions, marked by yellow circles in panels f and i of Figure 1, indicate the presence of Co atoms. This is further corroborated by the atomic line profiles (Figure 1k) corresponding to the line scans indicated in Figure 1f. Electron energy loss spectroscopy (EELS) analysis (Figure 1j) of the area circled in yellow in Figure 1i confirms the less bright spots as Co atoms. The reduced contrast around these Co atoms, attributed to oxygen atoms, suggests the formation of oxygen vacancies in the vicinity of the Co dopants, forming Co–O­(V) motifs. This observation aligns with stoichiometric considerations. The presence of oxygen vacancies is known to influence catalyst electronic structure, impacting electronic density of states, d-band center position, and band gap.^36,37^ Elemental mapping (Figure 1l) demonstrates the uniform distribution of Ru, Co, and O elements in Co_0.108_–RuO_2_, confirming the homogeneous incorporation of Co within the RuO_2_ lattice without Co agglomeration.

The influence of Co doping on RuO_2_ structure was investigated by comparing the morphology and composition of pure RuO_2_, Co_0.087_–RuO_2_, and Co_0.108_–RuO_2_ synthesized using the same method. STEM imaging (panels a–c of Figure 2) revealed a decrease in nanosheet grain size with increasing Co content: from 12.2 ± 2.28 nm for pure RuO_2_ to 3.08 ± 0.46 nm for Co_0.087_–RuO_2_ and 2.86 ± 0.49 nm for Co_0.108_–RuO_2_. This result is in agreement with the AFM thickness results for RuO_2_ and Co_0.083_–RuO_2_ (Figure S2). EELS analysis (Figure 2d) confirmed the correlation between decreasing grain size and increasing Co content, suggesting that Co doping effectively reduces RuO_2_ grain size, thereby increasing the specific surface area. XPS was employed to analyze the electronic structure and valence states of the Co_0.108_–RuO_2_, Co_0.083_–RuO_2_ catalyst, comparing it to pure RuO_2_ and commercial RuO_2_. Full XPS spectra (Figure S7a) confirmed the presence of Co within the RuO_2_ matrix. The Ru 3d and C 1s XPS spectra (Figure 2e) of Co_0.108_–RuO_2_ show peaks at 284.6 and 287.66 eV, attributed to C–C and C=O bonds, respectively. Peaks at 280.69 and 281.95 eV correspond to Ru 3d_5/2_ states for Ru^0^ and Ru^4+^, respectively.^38^ These Ru peaks exhibit a negative shift compared to those of pure RuO_2_. Further analysis of the Ru 3p orbital (Figure 2f) reveals peaks at 462.84 and 465.8 eV, assigned to Ru^0^ (3p_3/2_) and Ru^4+^ (3p_3/2_), respectively, and peaks at 484.82 and 487.83 eV corresponding to Ru^0^ (3p_1/2_) and Ru^4+^ (3p_1/2_), respectively.^39^ These Ru 3p peaks also show a negative shift compared to RuO_2_. This negative shift in both Ru 3d and Ru 3p spectra was consistently observed across different Co doping concentrations (panels b and c of Figure S7), confirming the consistent influence of Co doping on RuO_2_ electronic structure. This result indicates that there is greater charge transfer to Ru, resulting in increased charge distribution around Ru and lower Ru valence states. The Co 2p spectrum of Co_0.108_–RuO_2_ (Figure 2g) exhibits peaks at 779.38, 781.92, and 787.23 eV, corresponding to Co^2+^, Co^3+^, and a Co satellite peak for Co 2p_3/2_, respectively. Peaks at 795.3 and 796.6 eV are assigned to Co^2+^ and Co^3+^ states of Co 2p_1/2_, respectively.^40^ Compared to Co_3_O_4_, the Co 2p peaks in Co_0.108_–RuO_2_ are positively shifted, and a new satellite peak appears. This trend is consistent across different Co doping levels (Figure S7d). These observations suggest charge transfer from Co to Ru, which complements the negative shift in Ru spectra and leads to charge enrichment around Ru, thereby lowering its valence state.^41,42^ Analysis of the O 1s spectra (Figure 2h and Figure S8a) reveals three oxygen species. In Co_0.108_–RuO_2_ and Co_0.087_–RuO_2_, the peak at 529.16 eV is attributed to the Ru–O lattice bond, the peak at 532.27 eV to surface C=O bonds, and the peak at 530.41 eV to oxygen vacancies (O_V_).^26^ The increased intensity of the O_V_ peak in Co_0.108_–RuO_2_ and Co_0.087_–RuO_2_ compared to RuO_2_ suggests a higher concentration of oxygen vacancies. By calculating the O_V_ content of each catalyst (Table S2), it was determined that the O_V_ content of Co_0.108_–RuO_2_ was optimal. This, coupled with the negative shift in Ru 3p binding energies compared to commercial RuO_2_, further supports the presence of oxygen vacancies and their critical role in facilitating electron transfer, which contributes to lowering the Ru valence state. Specifically, oxygen vacancies enhance electron redistribution by acting as electron donors, weakening the Ru–O bond strength.^42^ EPR spectroscopy provided further evidence for oxygen vacancies. Co_0.108_–RuO_2_ and other Co_*x*_–RuO_2_ samples exhibit symmetrical peaks around *g* = 2.003 (Figure 2i and Figure S8b), indicative of unpaired electrons associated with oxygen vacancies.^43^ The weak EPR signal of commercial RuO_2_ confirms its low concentration of oxygen vacancies. In conclusion, the presence of oxygen vacancies in Co-doped RuO_2_, along with the observed negative shifts in Ru 3d and 3p binding energies and the positive shift in Co 2p spectra, suggests a synergistic electron redistribution and transfer process. Charge is transferred from Co to Ru, facilitated by oxygen vacancies, which weakens the Ru–O bond and stabilizes lower Ru valence states, enhancing the electronic properties of the material.

**Figure 2:**
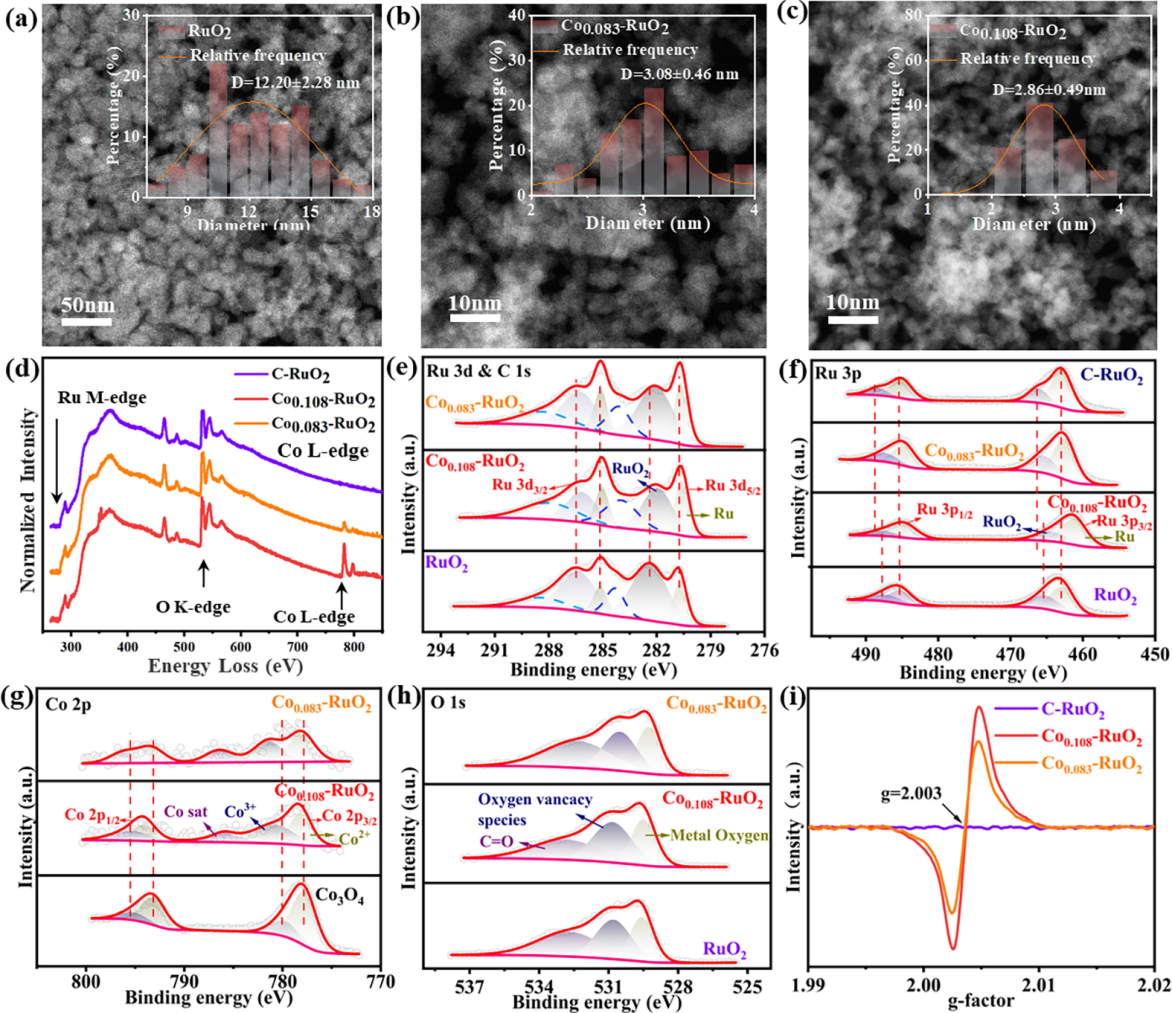
(a) STEM image and diameter distribution (inset) of RuO_2_. (b) STEM image and diameter distribution (inset) of Co_0.083_–RuO_2_. (c) STEM image and diameter distribution (inset) of Co_0.108_–RuO_2_. (d) EELS of the Co L-edge for RuO_2_, Co_0.083_–RuO_2_, and Co_0.108_–RuO_2_. (e) High-resolution X-ray photoelectron spectra of Ru 3d and C 1s of RuO_2_, Co_0.083_–RuO_2_, and Co_0.108_–RuO_2_. (f) High-resolution X-ray photoelectron spectra of Ru 3p of C–RuO_2_, RuO_2_, Co_0.083_–RuO_2_, and Co_0.108_–RuO_2_. (g) High-resolution X-ray photoelectron spectra of the Co 2p of Co_3_O_4_, Co_0.083_–RuO_2_, and Co_0.108_–RuO_2_. (h) High-resolution X-ray photoelectron spectra of O 1s for RuO_2_, Co_0.083_–RuO_2_, and Co_0.108_–RuO_2_. (i) EPR spectra of C–RuO_2_, Co_0.083_–RuO_2_, and Co_0.108_–RuO_2_.

To evaluate the OER electrocatalytic performance of Co_0.108_–RuO_2_, a three-electrode system was employed for electrochemical testing. The electrolyte was selected as 0.5 M H_2_SO_4_, the Hg/Hg_2_SO_4_ electrode was selected as a reference electrode, the Pt electrode (1 × 1 cm) was selected as a counter electrode, and the catalyst-coated carbon paper with a surface area of 1 cm^2^ was utilized as the working electrode. The catalyst loading on the carbon paper was 0.282 mg cm^–2^. The linear scanning voltammetric curves of Co_*x*_–RuO_2_ samples with varying molar concentrations were compared with those of RuO_2_ and commercial RuO_2_ samples (Figures 3a and panels a and b of Figure S9). The performance of the Co_*x*_–RuO_2_ samples was found to be superior to that of the RuO_2_ samples, with Co_0.108_–RuO_2_ exhibiting the best performance. Specifically, Co_0.108_–RuO_2_ exhibits the lowest peak starting potential, with an overpotential of 214 mV at a current density of 10 mA cm^–2^. This value is significantly lower than that of RuO_2_ (248 mV) and commercial RuO_2_ (318 mV), confirming its superior OER activity. The mass and specific activities were calculated by normalizing the current to the catalyst loading, surface area, and electrochemically active surface area (ECSA) at the working electrode.^17^ The mass and specific activities of the Co_*x*_–RuO_2_ samples and RuO_2_ were evaluated at 1.50 V [reversible hydrogen electrode (RHE)], where the mass and specific activities of Co_0.108_–RuO_2_ were 101.98 mA mg^–1^_oxide_ and 0.149 mA cm^–2^_oxide_, respectively, both of which exceeded those of RuO_2_ (52.16 mA mg^–1^_oxide_ and 0.118 mA cm^–2^_oxide_) and commercial RuO_2_ (18 mA mg^–1^_oxide_ and 0.11057 mA cm^–2^_oxide_) (Figure 3b and panels a, b, and d of Figure S10). Additionally, LSV curves normalized by the ECSA also revealed that Co_0.108_–RuO_2_ possesses the highest intrinsic activity (Figure S10c). The results indicated that Co_0.108_–RuO_2_ demonstrated the optimal intrinsic OER activity and the doping of the Co element did not reduce the catalytic activity of the RuO_2_ matrix. The Tafel slope is an effective indicator for checking the microkinetics of catalytic reactions.^44^ A comparative analysis of the Tafel plots of Co_*x*_–RuO_2_, RuO_2_, and commercial RuO_2_ was conducted (Figure 3c and Figure S9c). The Tafel slope for Co_0.108_–RuO_2_ was determined to be 78 mV dec^–1^, slightly lower than other Co_*x*_–RuO_2_ samples but significantly lower than both pure RuO_2_ (97 mV dec^–1^) and commercial RuO_2_ (131 mV dec^–1^). This difference in Tafel slopes suggests that Co doping alters the rate-determining step in the OER process. Consequently, Co_0.108_–RuO_2_ exhibits the lowest overpotential when the catalyst is required to achieve the same density. To gain insight into the origin of the OER activity, we performed a comparative analysis of the roughness factor (*R*_f_) and the electrochemically active area (ECSA) by calculating the electrical double-layer capacitance (*C*_dl_) (Figures S9d and S11 and Table S4). As shown in Figure 3d, the *C*_dl_ of RuO_2_ (21.4 mF cm^–2^) and commercial RuO_2_ (7.85 mF cm^–2^) was significantly smaller than that of Co_0.108_–RuO_2_ (33.5 mF cm^–2^). In general, a high *R*_f_ value is indicative of a catalyst with a large electrochemically active surface area.^45^ According to the formula *R*_f_ = *C*_dl_/*C*_s_ (where *C*_s_ is the specific capacitance, which is usually equal to 0.06 mF cm^–2^),^46^ compared to RuO_2_ (*R*_f_ = 356.7) and commercial RuO_2_ (*R*_f_ = 130.8), Co_0.108_–RuO_2_ has a *R*_f_ value of 558.3. The ECSA is calculated by multiplying the *R*_f_ value by the geometric area (1 cm^2^) of the electrode. The surface area of Co_0.108_–RuO_2_ catalyst was 195.8 m^2^ g^–1^, which was larger than that of RuO_2_ (125.2 m^2^ g^–1^) and commercial RuO_2_ (45.9 m^2^ g^–1^). The aforementioned results are in agreement with the specific surface area as measured by BET (Figure S5). According to the recent literature, catalysts with two-dimensional nanosheet structures typically exhibit a larger specific surface area.^47^ The larger ECSA indicates that the Co_0.108_–RuO_2_ electrocatalyst has more OER catalytic active sites.^33^ The impedance of Co_*x*_–RuO_2_, RuO_2_, and commercial RuO_2_ was then evaluated by EIS. The Nyquist plots for each catalyst (Figure 3e and Figure S9e) were evaluated at 1.456 V (versus RHE), where the diameter of the semicircle correlates with the magnitude of the charge transfer resistance (*R*_ct_).^48^ The solution resistance (*R*_sol_) of all electrodes was about 1.3 Ω. The Co_0.108_–RuO_2_ electrode had the smallest semicircle diameter, resulting in the lowest charge transfer resistance (*R*_ct_) of about 3.21 Ω. The results demonstrate that Co_0.108_–RuO_2_ exhibits the lowest interfacial resistance and the most rapid charge transfer rate, thereby enabling the most expeditious reaction kinetics and the highest intrinsic activity in the OER process. In order to determine the oxygen evolution efficiency of the OER process, the FE of the catalyst was calculated. The FE of Co_0.108_–RuO_2_ and Co_0.083_–RuO_2_ was comparatively analyzed by assembling the device as shown in Figure S13c. The results, as shown in panels a and b of Figure S13a and Table S3, showed that the FE of Co_0.108_–RuO_2_ is very closed to 100%. It shows that the side reactions have a negligible effect on the results. While high catalytic activity is undoubtedly a crucial factor, catalyst stability is of greater importance for practical applications. Accordingly, the stability of the catalyst was assessed through the chronopotentiometric method (*V*–*t*). Co_0.108_–RuO_2_ displayed notable stability following 45 h of operation at a current density of 10 mA cm^–2^ (Figure 3f). In contrast, both RuO_2_ and commercial RuO_2_ exhibited a notable increase in overpotential and eventual deactivation after stability testing. To verify that Co-doping can effectively prevent the lattice oxidation of RuO_2_ in OER, we conducted stability tests on Co_*x*_–RuO_2_ with varying molar concentrations. Co_*x*_–RuO_2_ exhibited markedly enhanced stability in comparison to RuO_2_ (Figure S9f), indicating that the Co element can effectively modify RuO_2_ and modulate the geometrical and electronic structures of the substrate, thereby exhibiting excellent OER stability. Compared to commercial IrO_2_, Co_0.108_–RuO_2_ also has the best catalytic activity and stability (Figure S12). Furthermore, Co_0.108_–RuO_2_ showed the lowest overpotential at a current density of 10 mA cm^–2^ (Figure 3g and Table S5), which surpassed the performance of previously reported Ru-based electrocatalysts under acidic conditions.

**Figure 3:**
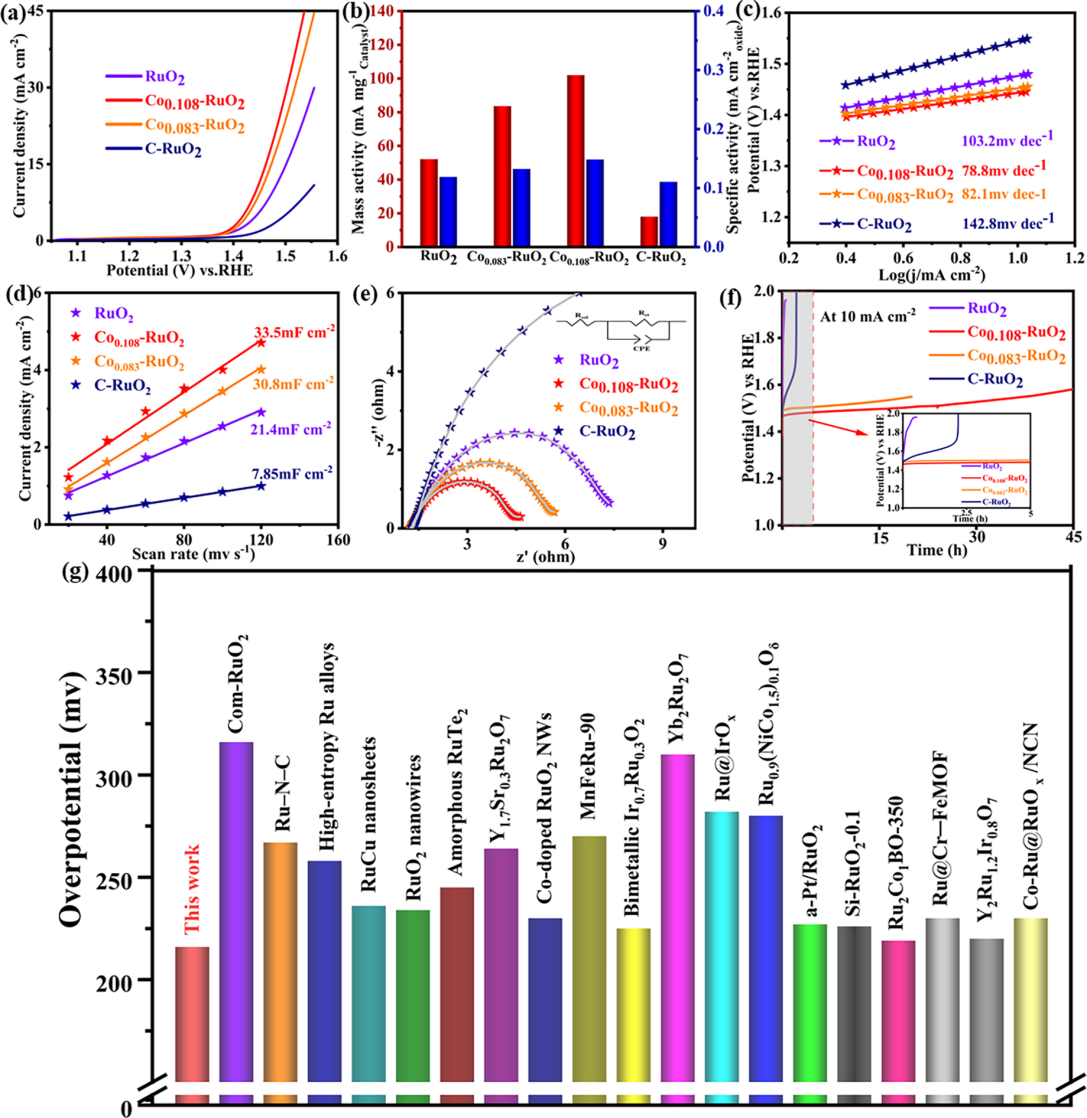
Electrochemical performances of RuO_2_, Co_0.083_–RuO_2_, Co_0.108_–RuO_2_, and C–RuO_2_ in an acidic medium (0.5 M H_2_SO_4_). (a) OER polarization curves. (b) Comparison of mass activity and specific activity at 1.5 V (versus RHE). (c) Tafel plots. (d) *C*_dl_ plots. (e) EIS plots. (f) Chronopotential test plots at 10 mA cm^–2^. (g) Comparison of the overpotential of Co_0.108_–RuO_2_ with that of a recently reported Ru-based electrocatalyst at a current density of 10 mA cm^–2^ in an acidic medium.

The stability of Co_0.108_–RuO_2_ and RuO_2_ was assessed after a 10 h chronopotentiometry test. Post-test characterization using XRD, TEM, and energy-dispersive X-ray spectroscopy (EDX) mapping (panels a–c of Figure 4) confirmed the structural integrity of Co_0.108_–RuO_2_ and the continued presence of Co, demonstrating its excellent geometric stability. To understand the origin of this enhanced stability, the OER mechanism was investigated. Typically, the oxygen evolution reaction can proceed via two primary pathways: the AEM involving a concerted proton–electron transfer step, and the LOM characterized by non-concerted proton–electron transfer and pH-dependent catalytic activity.^49–52^ As shown in Figure 4d and Figure S14, the catalytic activity of Co_0.108_–RuO_2_ displayed negligible pH dependence, while RuO_2_ exhibited a strong pH dependence. This suggests that Co doping may promote a shift in the OER mechanism from LOM to AEM. Changes in the Ru 3p chemical state before and after the stability test were also examined (Figure 4e). A slight positive shift in the Ru 3p peak of Co_0.108_–RuO_2_ post-test indicates some degree of oxidation, which is expected at high anodic potentials. However, the Ru 3p peaks of RuO_2_ exhibited a more pronounced positive shift, suggesting a greater extent of oxidation. This indicates that Co doping effectively mitigates Ru oxidation during the OER in an acidic medium. Subsequently, the oxygen evolution was elucidated based on the O 1s spectra obtained before and after the stability test. The O_V_/O_L_ ratios of RuO_2_ were significantly increased (1.37–2.33) (Figure 4f and Figure S15a), indicating that the lattice oxygen played an important role in the generation of O_2_ by accelerating the crystal structure collapse and the dissolution of active Ru in RuO_2_^1^. In contrast, the Ov/O_L_ ratio for Co_0.108_–RuO_2_ only slightly increased (from 1.41 to 1.47) (Figure 4f and Figure S15b), supporting the dominance of the AEM pathway over the LOM pathway. EPR analysis corroborated these findings. The intensity of the oxygen vacancy signal (around *g* = 2.003) remained essentially unchanged for Co_0.108_–RuO_2_ after the stability test (Figure 4g), while the corresponding signal for RuO_2_ increased significantly (inset of Figure 4g). This further confirms the prevalence of the AEM pathway for Co_0.108_–RuO_2_ and the LOM pathway for RuO_2_. Collectively, these results demonstrate that the enhanced activity and stability of Co_0.108_–RuO_2_ arise from the Co-induced shift in the OER mechanism from the less stable LOM pathway to the more stable AEM pathway.

**Figure 4:**
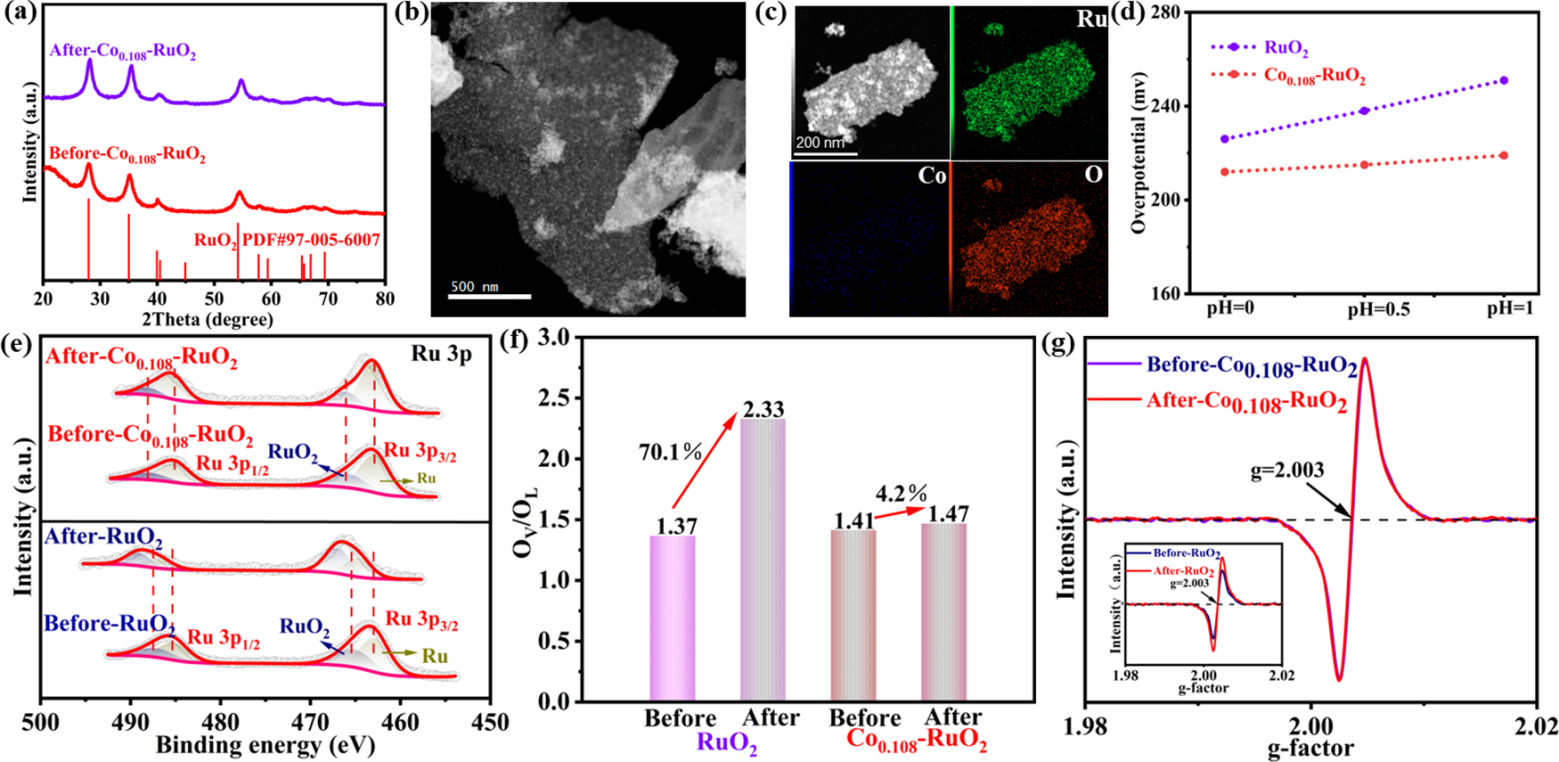
(a) XRD patterns of Co_0.108_–RuO_2_ both before and after the stability test. (b) STEM image of Co_0.108_–RuO_2_ following the stability test. (c) EDS energy spectrum after Co_0.108_–RuO_2_ stability test. (d) Overpotentials (at 10 mA cm^–2^) of Co_0.108_–RuO_2_ and RuO_2_ under varying pH conditions. (e) High-resolution X-ray photoelectron spectra of Ru 3p were obtained for Co_0.108_–RuO_2_ and RuO_2_ before and after the stability tests. (f) O_V_/O_L_ ratios of Co_0.108_–RuO_2_ and RuO_2_ before and after stability tests. (g) EPR spectra of Co_0.108_–RuO_2_ before and after the stability test, with the inset displaying the EPR spectra of RuO_2_ before and after the stability test.

Building upon the experimental observations, DFT calculations were performed to elucidate the impact of Co doping and oxygen vacancies on the OER mechanism. Four models based on the RuO_2_(110) surface were constructed (panels a–d of Figure 5) to simulate different active site configurations: RuO_2_–LOM (perfect surface), RuO_2_–O_V_–LOM (oxygen vacancies near the adsorption site), Co–RuO_2_–O_V_–LOM (Co substituted for Ru near oxygen vacancies), and Co–RuO_2_–O_V_–AEM (Co substitution and oxygen vacancies). The Co–RuO_2_–O_V_–AEM model specifically employed the AEM mechanism for free energy calculations, while the remaining models utilized the LOM mechanism, allowing for direct comparison of the two pathways. The aim was to determine the rate-determining steps (RDS) and associated energy barriers for each model, thereby providing a mechanistic understanding of the enhanced OER activity observed experimentally. The reaction energy barriers for the four model catalyst OER pathways with 0 V potential were calculated (panels e–h of Figure 5 and Figure S16 and Table S6). The theoretical calculations demonstrated that the RDS differed between the three models. The RDS of the Co–RuO_2_–O_V_–AEM model is an OOH* transition to an OO* intermediate, with a free energy barrier of 2.599 eV. The RDS of the Co–RuO_2_–O_V_–LOM is an O* transition to an OO* intermediate, with a free energy barrier of 2.905 eV. These findings are consistent with the experimental results, which indicate that the OER process in the AEM mechanism exhibits superior catalytic activity. The RDS of the RuO_2_–LOM model is the O* transition to OO* intermediate, which occurs with a free energy barrier of 3.428 eV. The RuO_2_–O_V_–LOM model is the OO* to OO* intermediate transition, which is a thermodynamic process transition^53^ that requires the overcoming of an energy barrier of 3.308 eV. The results indicate that when Co doping and O_V_ are present in the RuO_2_ matrix, the energy barrier necessary for RDS in the OER process can be reduced. The aforementioned calculations demonstrate that the doping of RuO_2_ with Co results in a conversion of the OER mechanism from LOM toAEM on the one hand, and the presence of O_V_ in the RuO_2_ matrix on the other hand, which, when combined, markedly enhances the catalytic performance of Co–RuO_2_–O_V_–AEM.

**Figure 5:**
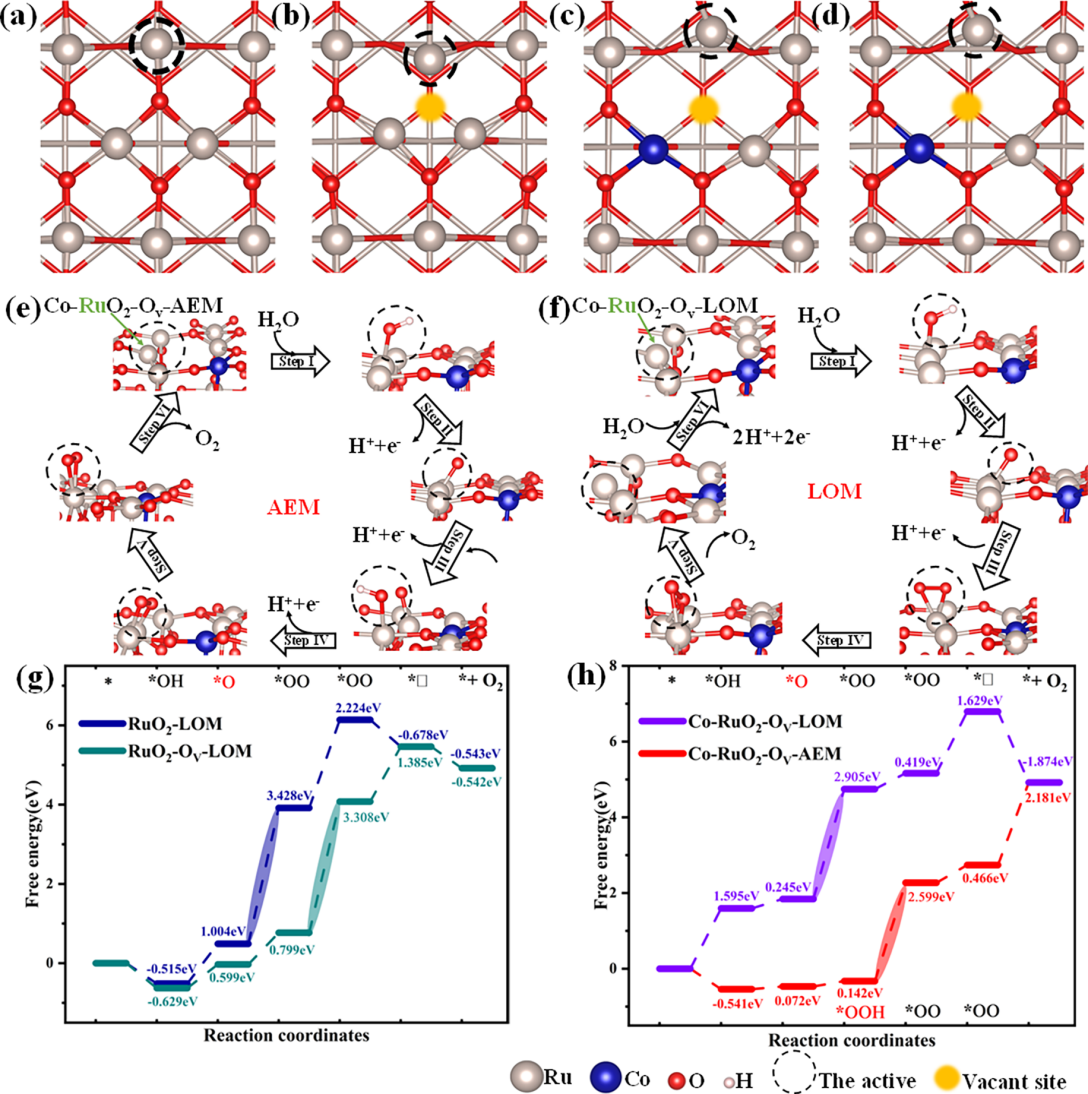
(a) Top view of RuO_2_–LOM. (b) Top view of RuO_2_–O_V_–LOM. (c) Top view of Co–RuO_2_–O_V_–LOM. (d) Top view of Co–RuO_2_–O_V_–AEM. (e) Co–RuO_2_–O_V_–AEM mechanism. (f) Co–RuO_2_–O_V_–LOM mechanism. (g) Energy barrier diagram for RuO_2_–LOM and RuO_2_–O_V_–LOM intermediates. (h) Energy barrier diagram for Co–RuO_2_–O_V_–LOM and Co–RuO_2_–O_V_–AEM intermediates.

## 4 Conclusion

In conclusion, this work demonstrates the successful synthesis of a porous RuO_2_ nanosheet structure composed of interconnected grains, where Co doping effectively modulates grain size, increasing both the specific surface area and the density of defects. Crucially, the incorporation of Co introduces oxygen vacancies, creating Co–O­(V) motifs that tune the electronic configuration of Ru. Experimental analyses demonstrate that this controlled introduction of oxygen vacancies reduces the Ru/O coordination ratio, effectively preventing the overoxidation of RuO_2_ to RuO_4_. These Co–O­(V) motifs drive a critical shift in the acidic OER mechanism from LOM to AEM, significantly enhancing the stability of the RuO_2_ matrix in acidic environments. DFT calculations confirm that the synergistic combination of a moderate amount of oxygen vacancies and the AEM pathway lowers the energy barriers for OER intermediates. As a result, the Co-doped RuO_2_ nanosheets exhibit superior OER performance, demonstrating the effectiveness of this strategy for developing robust and efficient electrocatalysts for challenging acidic conditions. This study highlights the importance of precisely engineered defects and electronic structure modulation in achieving enhanced electrocatalytic activity and durability.

## Supplementary Material



## Notes

XRD of RuO_2_ and Co_*x*_–RuO_2_, BET of RuO_2_ and Co_0.083_–RuO_2_, TEM of Co_0.108_–RuO_2_, N_2_ adsorption and desorption curves of RuO_2_, Co_0.083_–RuO_2_, Co_0.108_–RuO_2_, commercial RuO_2_, and commercial IrO_2_, pore size distribution of Co_0.108_–RuO_2_, XPS spectra of the comparison sample and Co_*x*_–RuO_2_, electrochemical performance and EPR spectra of the comparison sample and Co_*x*_–RuO_2_, FE and test setups of Co_0.083_–RuO_2_ and Co_0.108_–RuO_2_, DFT computational modeling and results, and comparisons of Co_0.108_–RuO_2_ to the rest of the literature reported η10 (PDF)
