# Supplementary material for: Co-Motif-Engineered RuO2 Nanosheets for Robust and Efficient Acidic Oxygen Evolution
Source: ACS Appl Mater Interfaces. 2025 Mar 19;17(13):19734–44. doi: 10.1021/acsami.5c00773 (PMC12755202; doi:10.1021/acsami.5c00773)
Supplement: Supplementary file 1 [file am5c00773_si_001.pdf]

Supporting Information

Co-Motif Engineered RuO_2 Nanosheets for Robust and Efficient Acidic Oxygen Evolution

Jiandong Hu[a‡], Le Tong[c‡], Yanlin Jia[a], Ziye Li[a], Haowei Yang[a], Yang Wang[a], Wenhui Luo[a], Yejun Li[a], Yong Pang[a], Shiyun Xiong[c], Zhi Liang Zhao[b]* and Qi Wang[d]**

[‡]: These authors have contributed equally to this work

[a] School of Materials Science and Engineering, Central South University, Changsha, Hunan 410083, P.R. China

[b] Foshan Xianhu Laboratory, National energy key laboratory for new hydrogen-ammonia energy technologies, Foshan, Guangdong 528200, P.R. China

[c] School of Materials and Energy, Guangdong University of Technology, Guangzhou 510006, P.R. China

[d] City University of Hong Kong, Department of Materials Science and Engineering, Hong Kong 999077, China

Yong Pang[a]* Email: thgink@126.com

Zhi Liang Zhao[b]* Email: zhaozhiliang@xhlab.cn

Qi Wang[d]* Email: qwang422@cityu.edu.hk

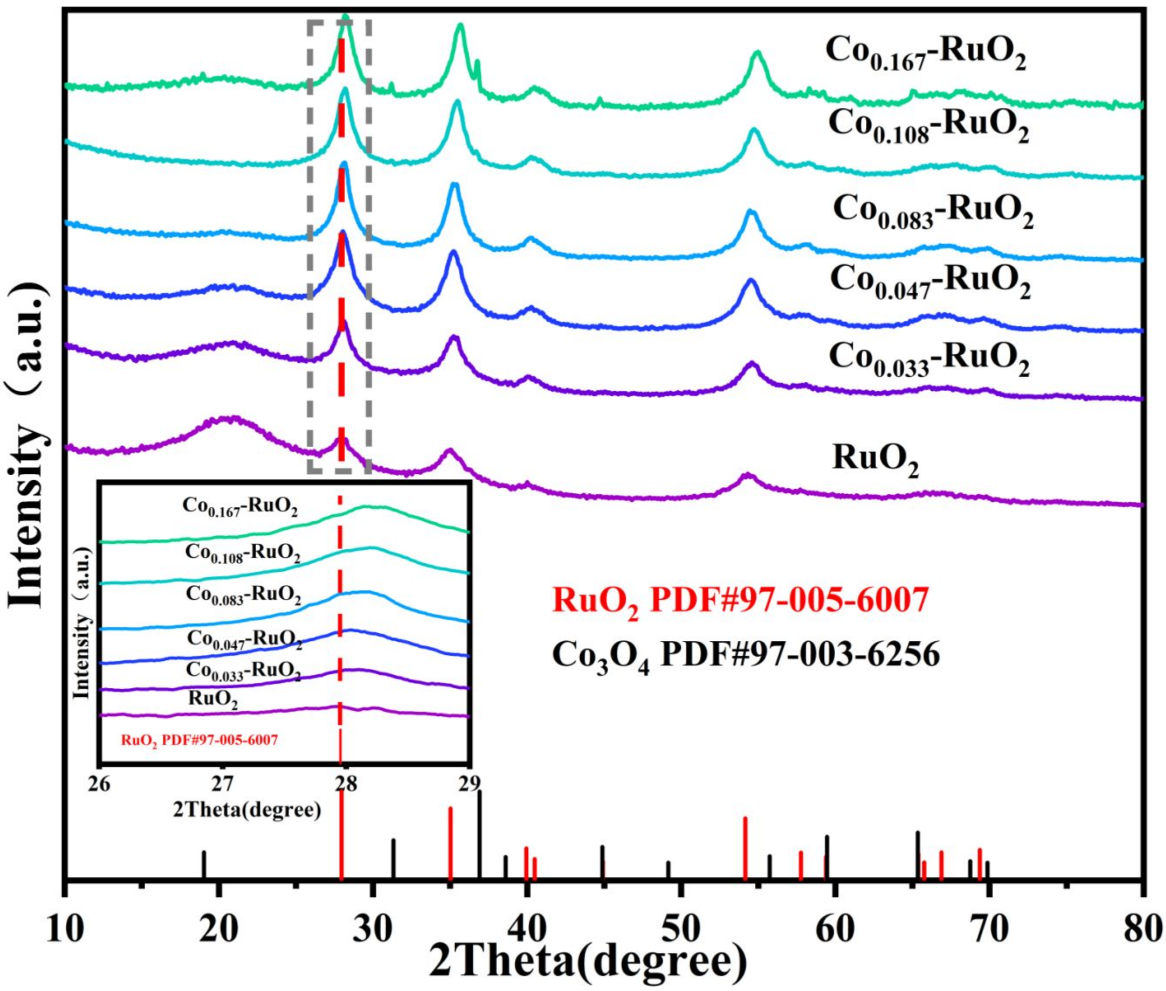

Figure S1. XRD spectra of RuO_2, $Co_{0.033}$-RuO_2, $Co_{0.047}$-RuO_2, $Co_{0.083}$-RuO_2, $Co_{0.108}$-RuO_2, and $Co_{0.167}$-RuO_2 (inset: magnified XRD spectra of the strongest peak of RuO_2, $Co_{0.033}$-RuO_2, $Co_{0.047}$-RuO_2, $Co_{0.083}$-RuO_2, $Co_{0.108}$-RuO_2, and $Co_{0.167}$-RuO_2, 26-29°).

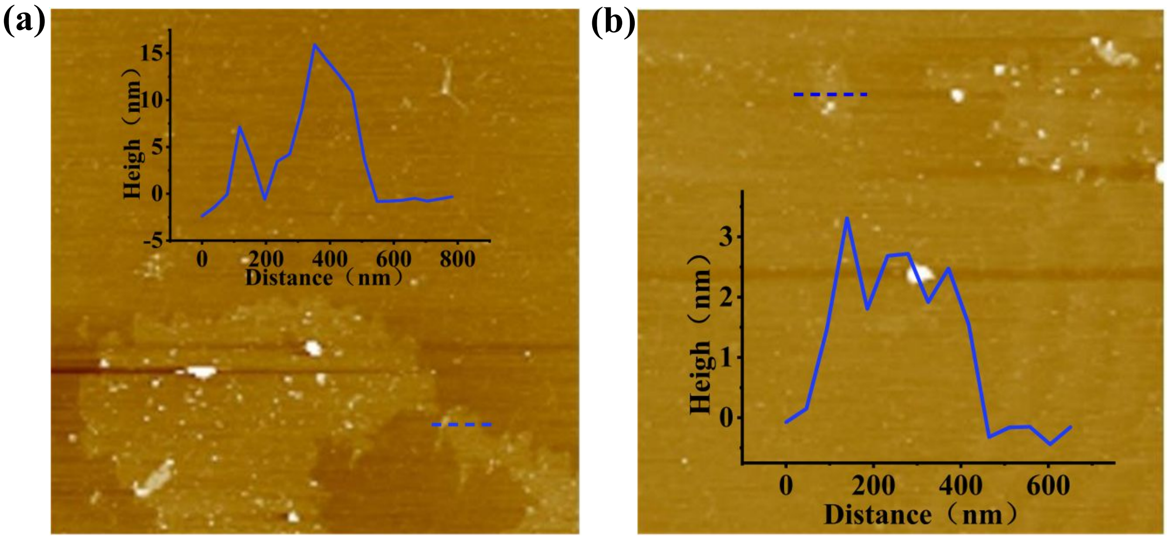

Figure S2. (a) AFM image of RuO_2. (b) AFM image of $Co_{0.083}$-RuO_2.

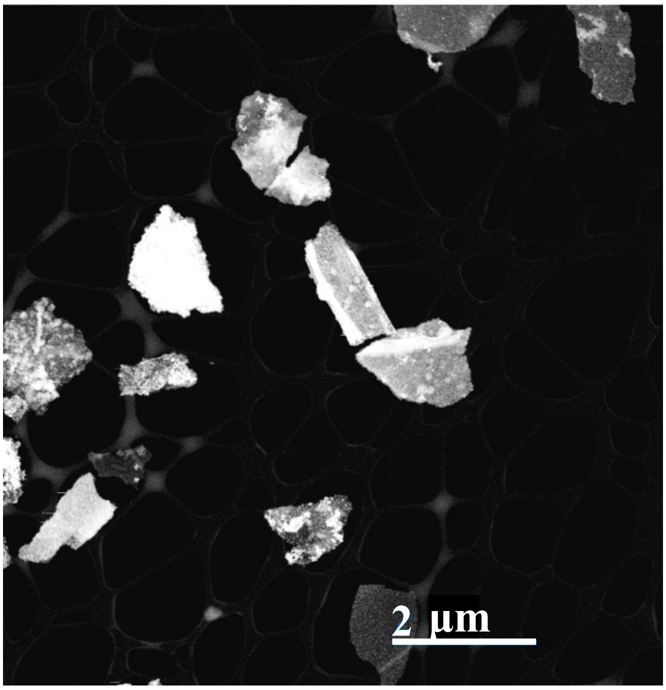

Figure S3. HR-STEM image of $Co_{0.108}$-RuO_2 (2 μm)

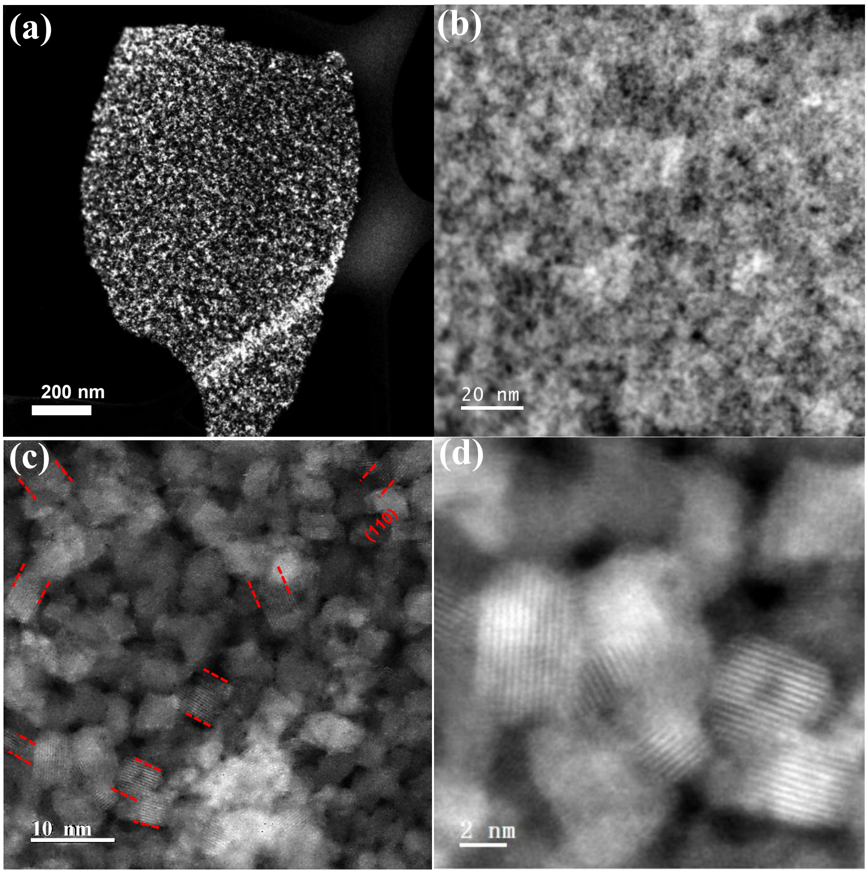

Figure S4. (a) High-magnification STEM image of $Co_{0.108}$-RuO_2 (200nm). (b) High-magnification STEM image of $Co_{0.108}$-RuO_2 (20nm). (c) High-magnification STEM image of $Co_{0.108}$-RuO_2 (10nm). (d) High-magnification STEM image of $Co_{0.108}$-RuO_2 (2nm).

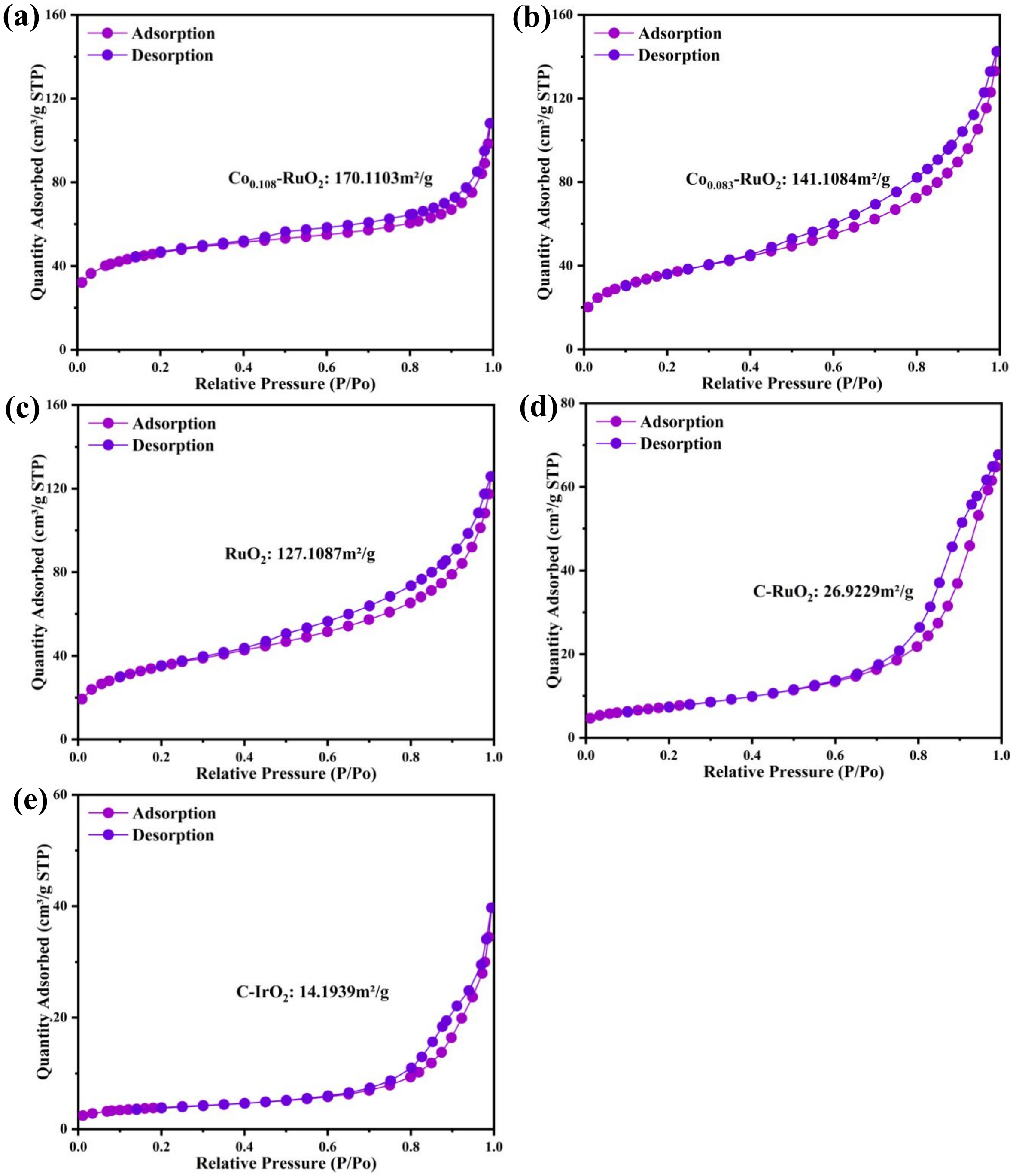

Figure S5. (a) The isotherm of N_2 adsorption and desorption on $Co_{0.108}$-RuO_2. (b) The isotherm of N_2 adsorption and desorption on $Co_{0.083}$-RuO_2. (c) The isotherm of N_2 adsorption and desorption on RuO_2. (d) The isotherm of N_2 adsorption and desorption on C-RuO_2. (e) The isotherm of N_2 adsorption and desorption on C-IrO_2.

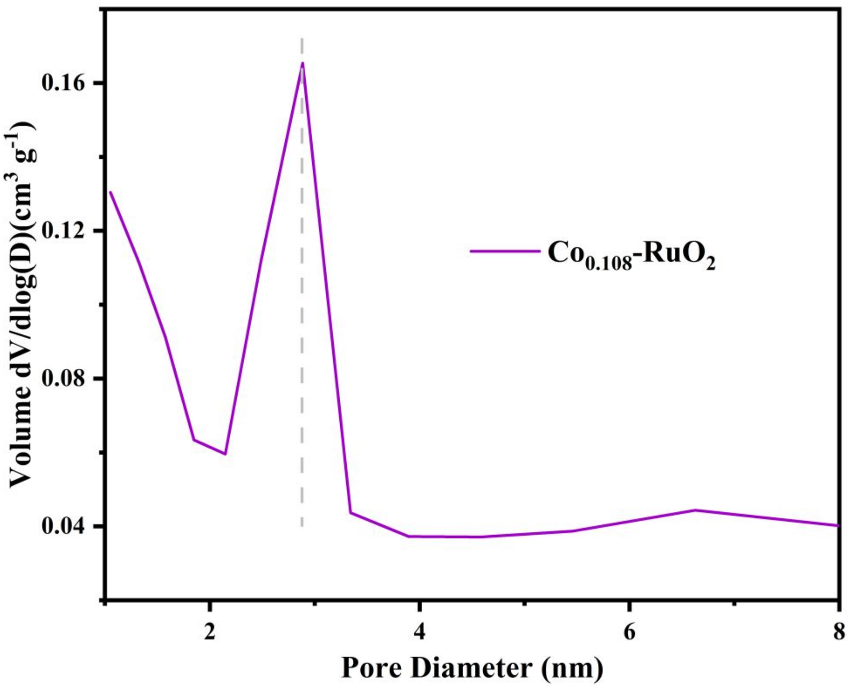

Figure S6. Pore size distribution of $Co_{0.108}$-RuO_2.

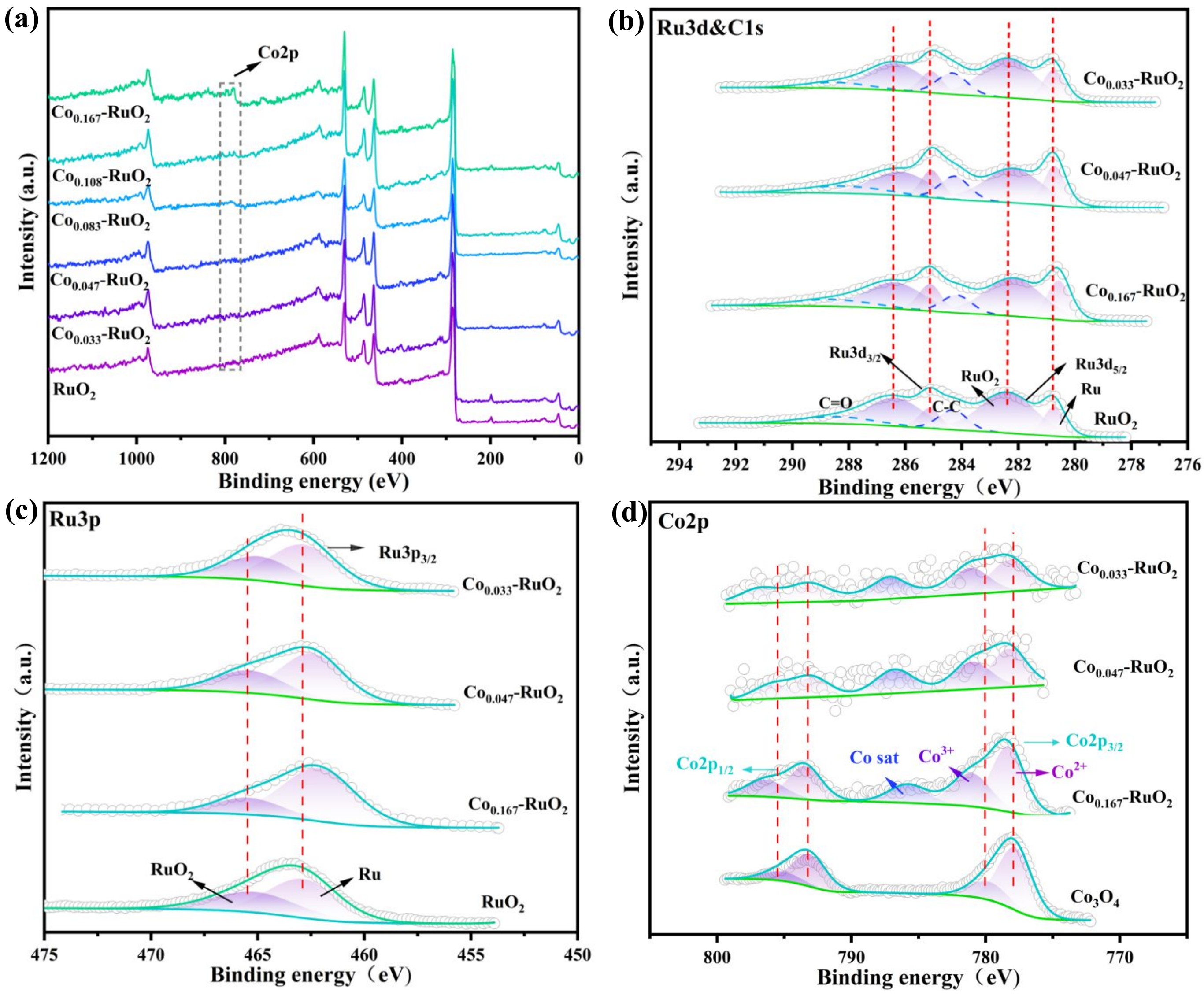

Figure S7. (a) The high-resolution XPS full-spectrum spectra of RuO_2, $Co_{0.033}$-RuO_2, $Co_{0.047}$-RuO_2, $Co_{0.083}$-RuO_2, $Co_{0.108}$-RuO_2, and $Co_{0.167}$-RuO_2. (b) High-resolution X-ray photoelectron spectra of Ru3d&C1s of RuO_2, $Co_{0.033}$-RuO_2, $Co_{0.047}$-RuO_2, and $Co_{0.167}$-RuO_2. (c) High-resolution X-ray photoelectron spectra of the Ru3p orbital of RuO_2, $Co_{0.033}$-RuO_2, $Co_{0.047}$-RuO_2, and $Co_{0.167}$-RuO_2. (d) High-resolution X-ray photoelectron spectra of Co2p of Co_3O_4, $Co_{0.033}$-RuO_2, $Co_{0.047}$-RuO_2, and $Co_{0.167}$-RuO_2.

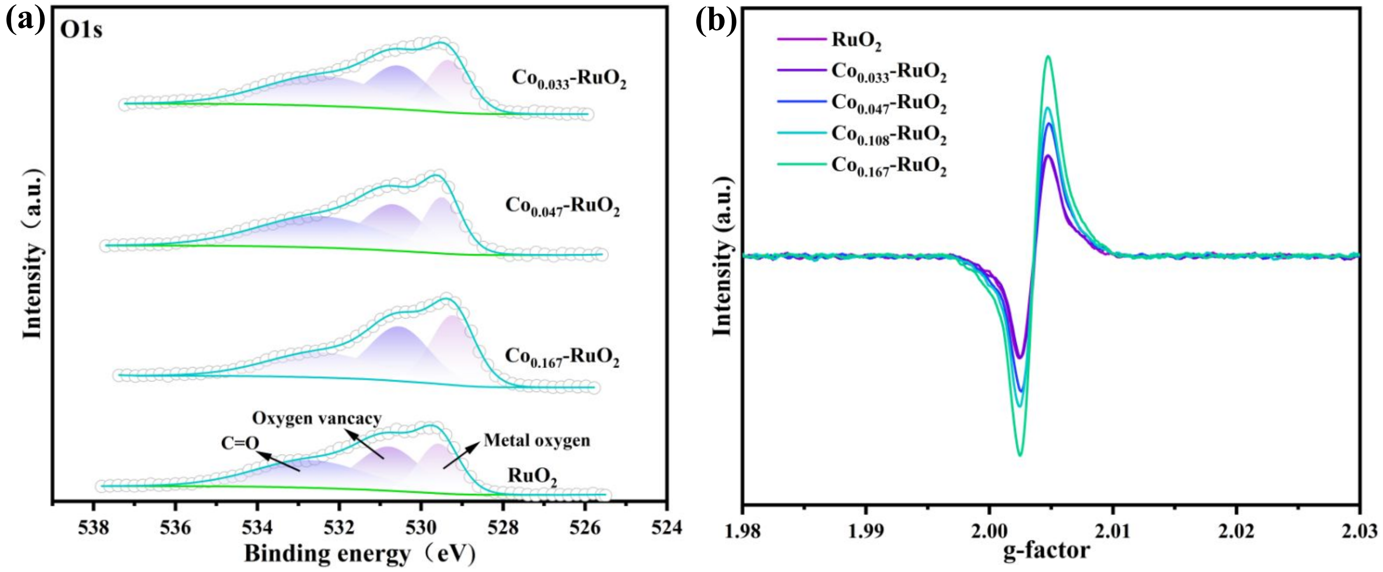

Figure S8. (a) High-resolution X-ray photoelectron spectra of O 1s for RuO_2, $Co_{0.033}$-RuO_2, $Co_{0.047}$-RuO_2, and $Co_{0.167}$-RuO_2. (b) EPR spectra of RuO_2, $Co_{0.033}$-RuO_2, $Co_{0.047}$-RuO_2, and $Co_{0.167}$-RuO_2.

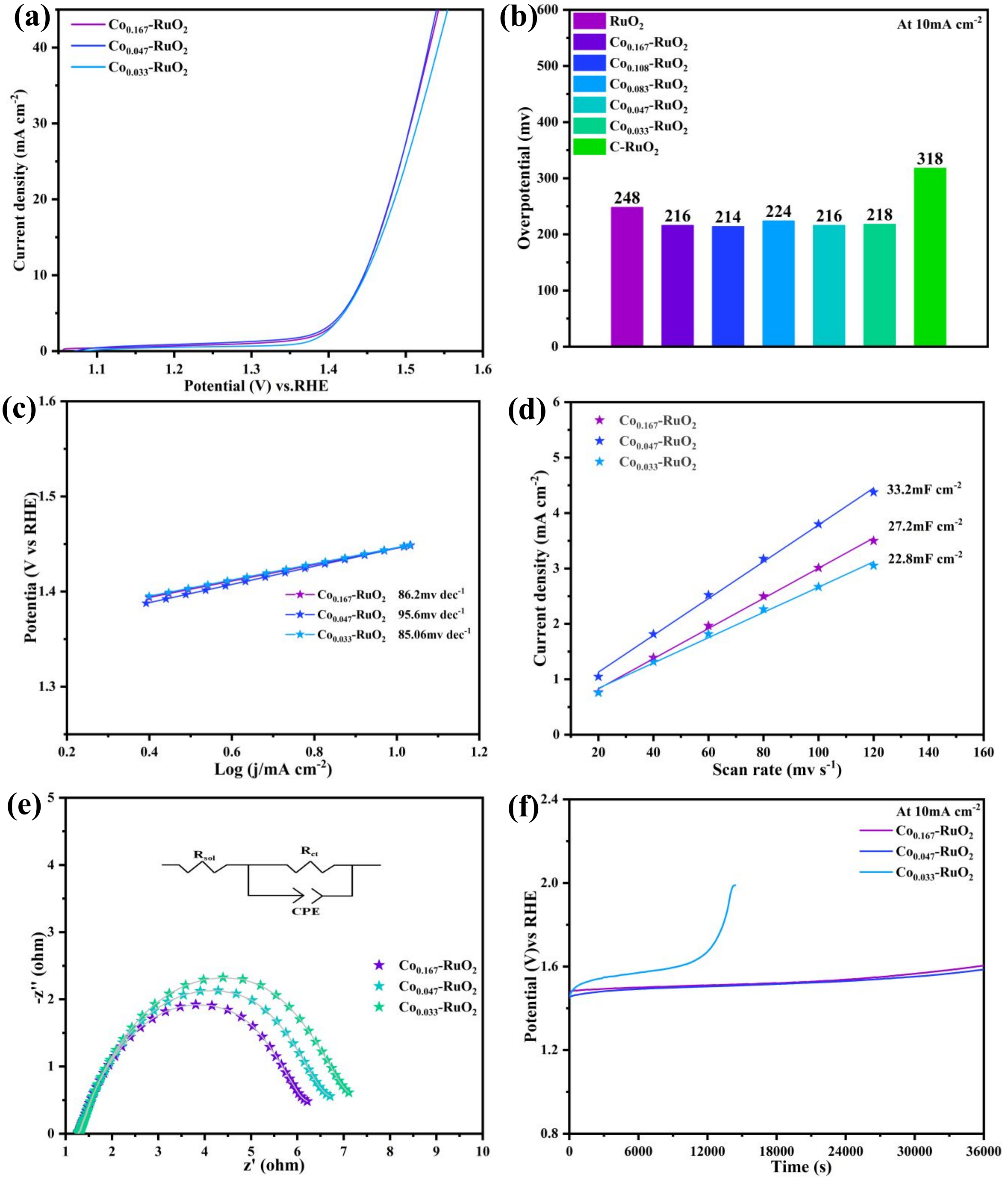

Figure S9. (a) Comparison of polarization curves of $Co_{0.033}$-RuO_2, $Co_{0.047}$-RuO_2, and $Co_{0.167}$-RuO_2. (b) Histogram of overpotentials of RuO_2, $Co_{0.033}$-RuO_2, $Co_{0.047}$-RuO_2, $Co_{0.083}$-RuO_2, $Co_{0.108}$-RuO_2, $Co_{0.167}$-RuO_2 and C-RuO_2 at 10 mA cm^{-2}. (c) Tafel plots for $Co_{0.033}$-RuO_2, $Co_{0.047}$-RuO_2, and $Co_{0.167}$-RuO_2. (d) C_{dl} plots for $Co_{0.033}$-RuO_2, $Co_{0.047}$-RuO_2, and $Co_{0.167}$-RuO_2. (e) EIS plots for $Co_{0.033}$-RuO_2, $Co_{0.047}$-RuO_2, and $Co_{0.167}$-RuO_2. (f) Chronopotential test plots for $Co_{0.033}$-RuO_2, $Co_{0.047}$-RuO_2, and $Co_{0.167}$-RuO_2.

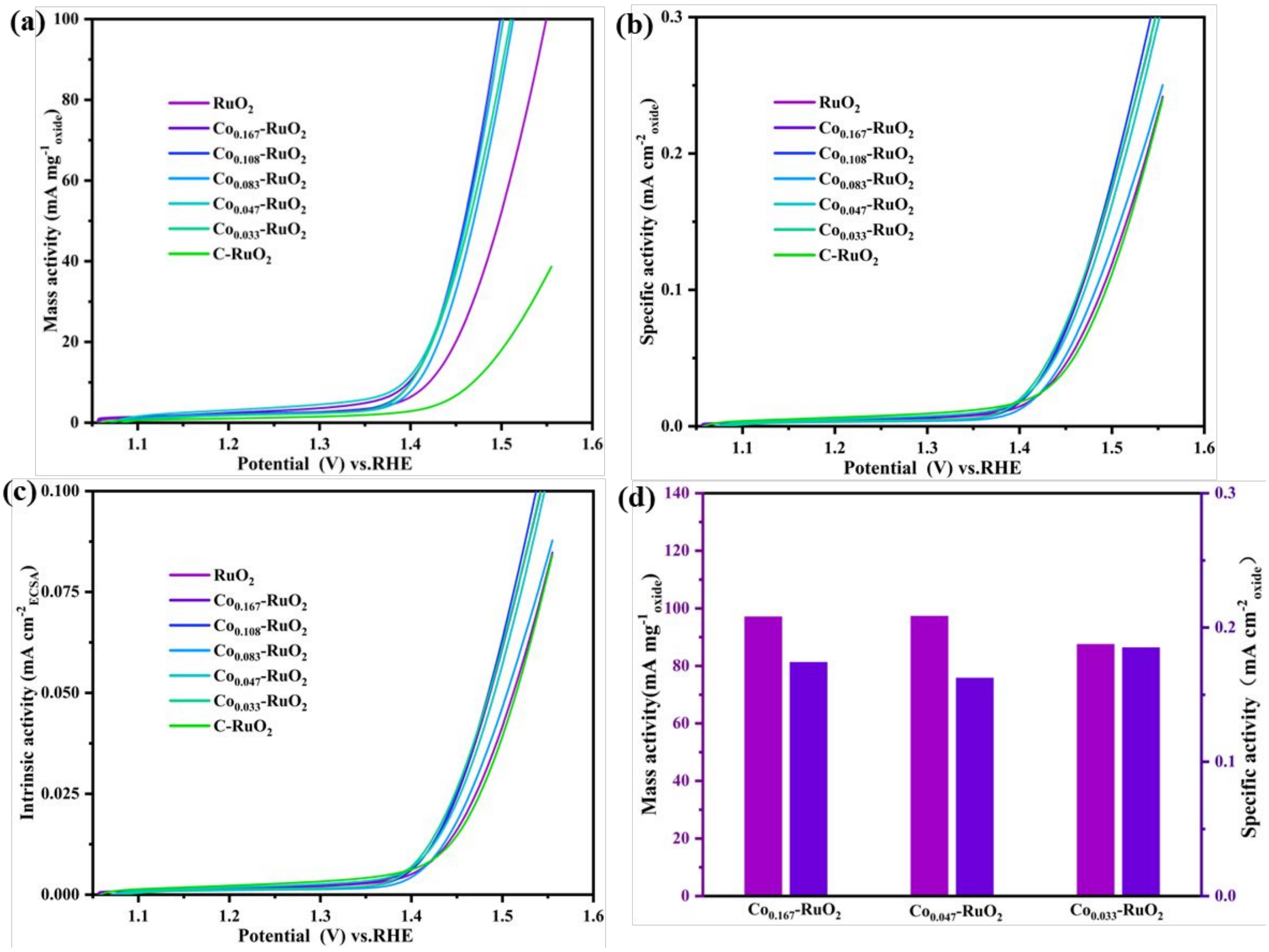

Figure S10. (a) Mass-normalised polarisation curves for RuO_2, $Co_{0.033}$-RuO_2, $Co_{0.047}$-RuO_2, $Co_{0.083}$-RuO_2, $Co_{0.108}$-RuO_2, $Co_{0.167}$-RuO_2, and C-RuO_2. (b) Normalized polarisation curves of specific surface area for RuO_2, $Co_{0.033}$-RuO_2, $Co_{0.047}$-RuO_2, $Co_{0.083}$-RuO_2, $Co_{0.108}$-RuO_2, $Co_{0.167}$-RuO_2, and C-RuO_2. (c). Normalized polarisation curves of ECSA for RuO_2, $Co_{0.033}$-RuO_2, $Co_{0.047}$-RuO_2, $Co_{0.083}$-RuO_2, $Co_{0.108}$-RuO_2, $Co_{0.167}$-RuO_2, and C-RuO_2. (d) Histograms of mass and specific activities of $Co_{0.033}$-RuO_2, $Co_{0.047}$-RuO_2, and $Co_{0.167}$-RuO_2 at 1.5 V (vs. RHE).

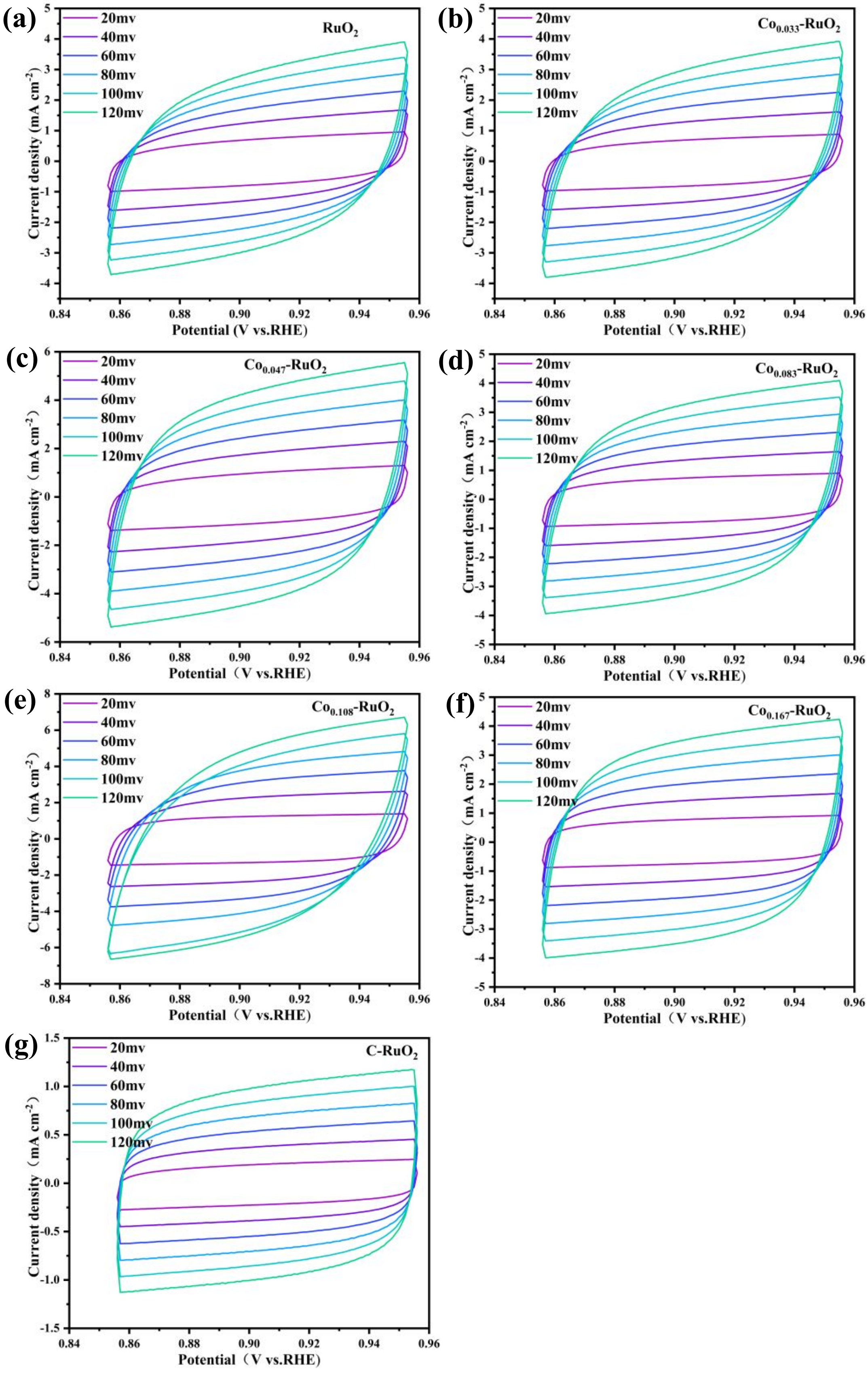

Figure S11. (a-g) Cyclic voltammetric curves for RuO_2, $Co_{0.033}$-RuO_2, $Co_{0.047}$-RuO_2, $Co_{0.083}$-RuO_2, $Co_{0.108}$-RuO_2, $Co_{0.167}$-RuO_2 and C-RuO_2 (20mv-120mv).

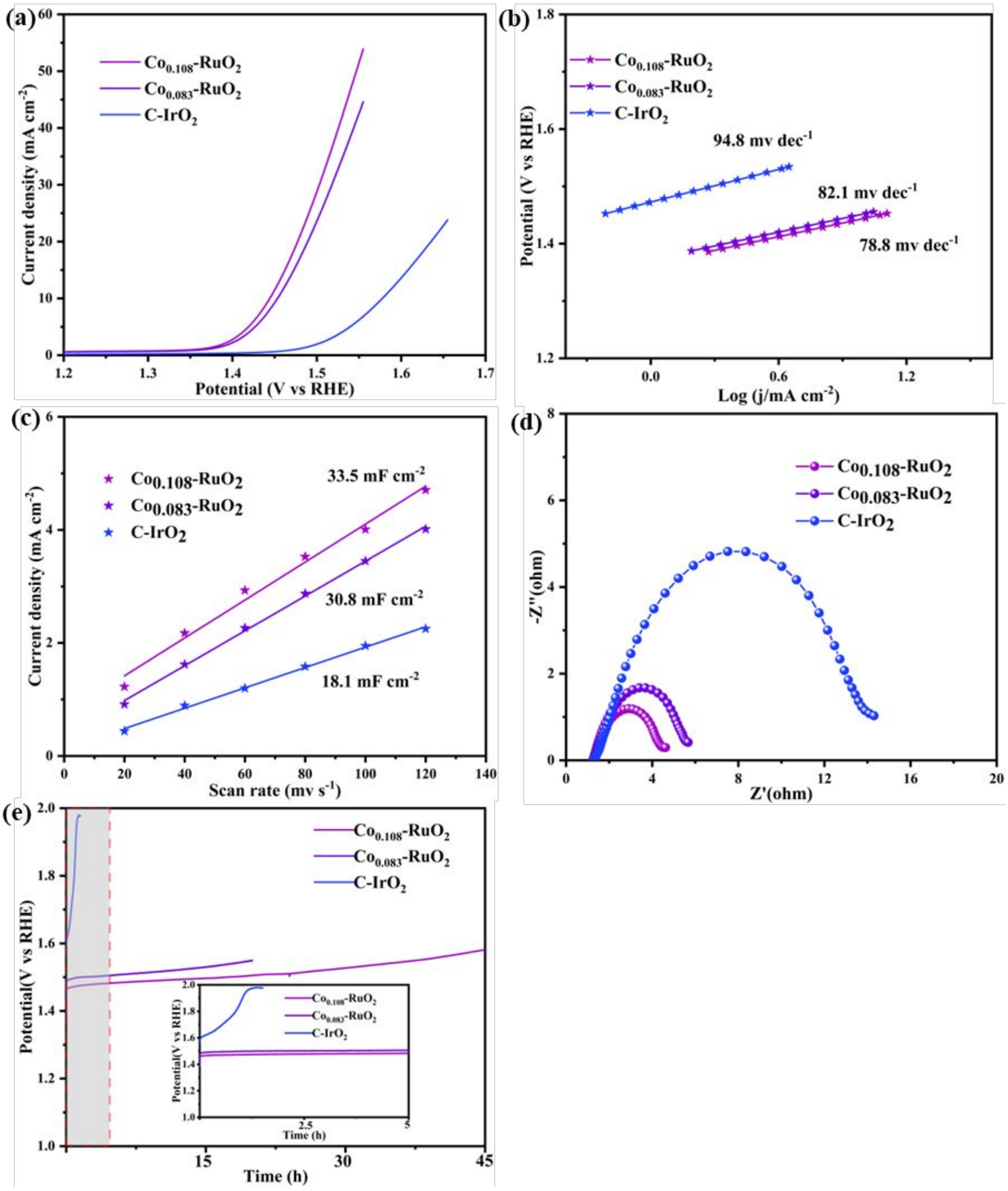

Figure S12. (a) Comparison of polarization curves of $Co_{0.108}$-RuO_2, $Co_{0.083}$-RuO_2, and C-IrO_2. (b) Tafel plots for $Co_{0.108}$-RuO_2, $Co_{0.083}$-RuO_2, and C-IrO_2. (c) C_{dl} plots for $Co_{0.108}$-RuO_2, $Co_{0.083}$-RuO_2, and C-IrO_2. (d) EIS plots for $Co_{0.108}$-RuO_2, $Co_{0.083}$-RuO_2, and C-IrO_2. (e) Chronopotential test plots for $Co_{0.108}$-RuO_2, $Co_{0.083}$-RuO_2, and C-IrO_2.

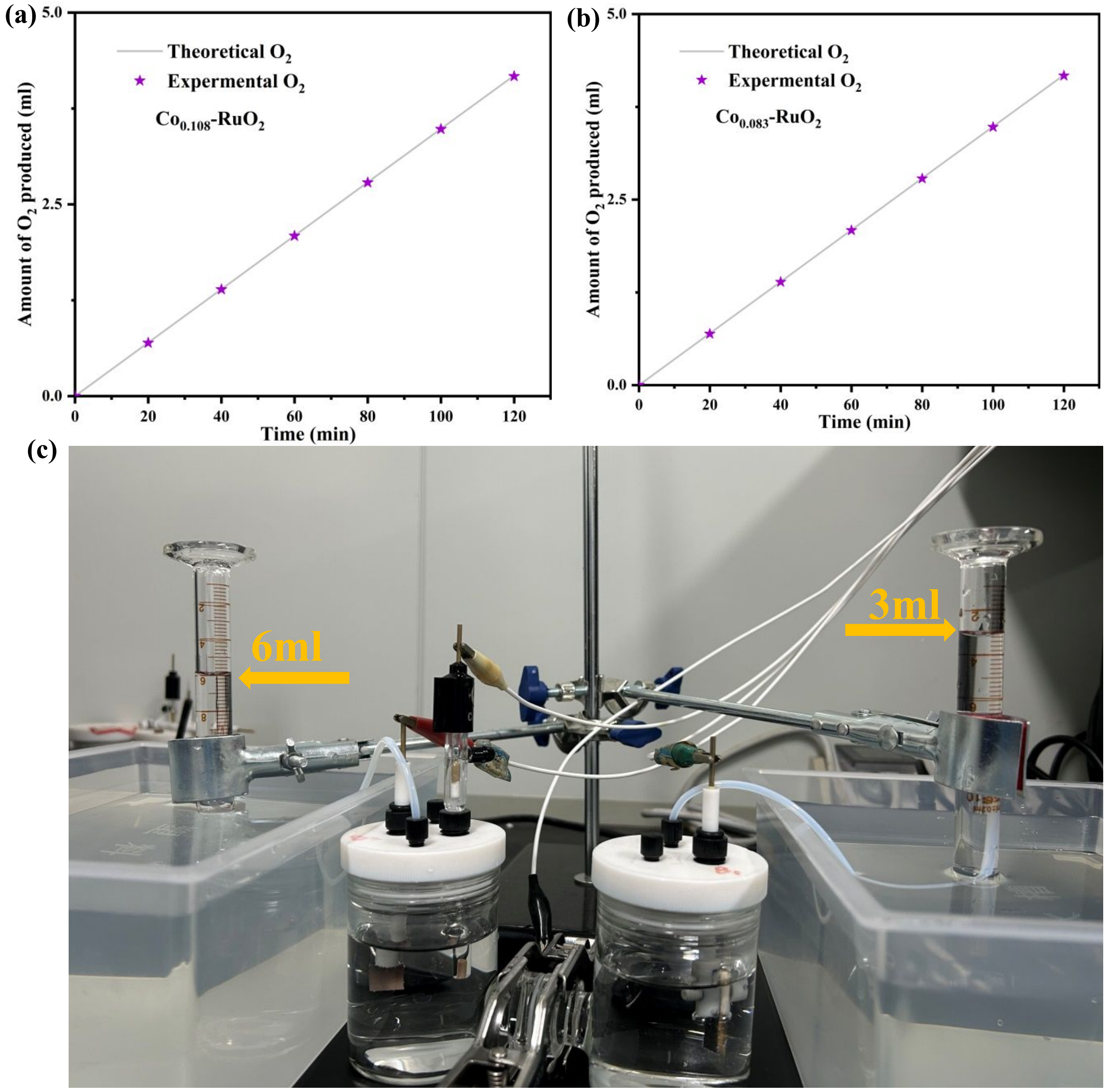

Figure S13. (a) Comparison of theoretically calculated and actual amounts of gas produced by $Co_{0.108}$-RuO_2 in 0.5 M H_2SO_4. (b) Comparison of the theoretically calculated and actual amounts of gas produced by $Co_{0.083}$-RuO_2 in 0.5 M H_2SO_4. (c) Diagram of the apparatus for testing the amount of oxygen produced by $Co_{0.108}$-RuO_2 and $Co_{0.083}$-RuO_2 in the OER process by the drainage method.

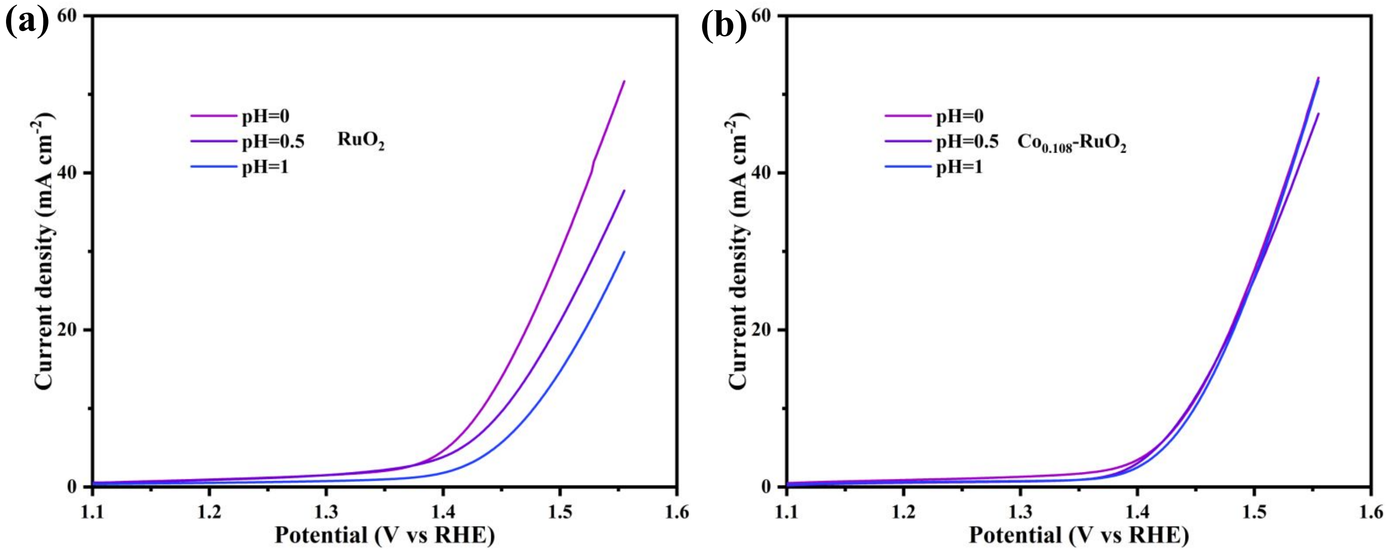

Figure S14. (a) Polarisation curves of RuO_2 at different pH values. (b) Polarisation curves of $Co_{0.108}$-RuO_2 at different pH values.

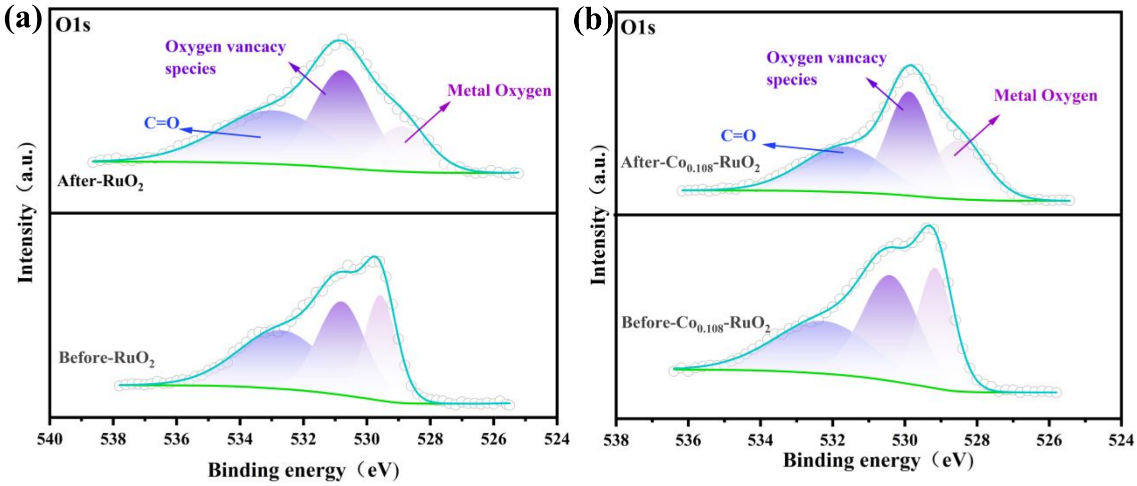

Figure S15. (a) XPS spectra of O1s before and after RuO_2 stability tests. (b) XPS spectra of O1s before and after $Co_{0.108}$-RuO_2 stability tests.

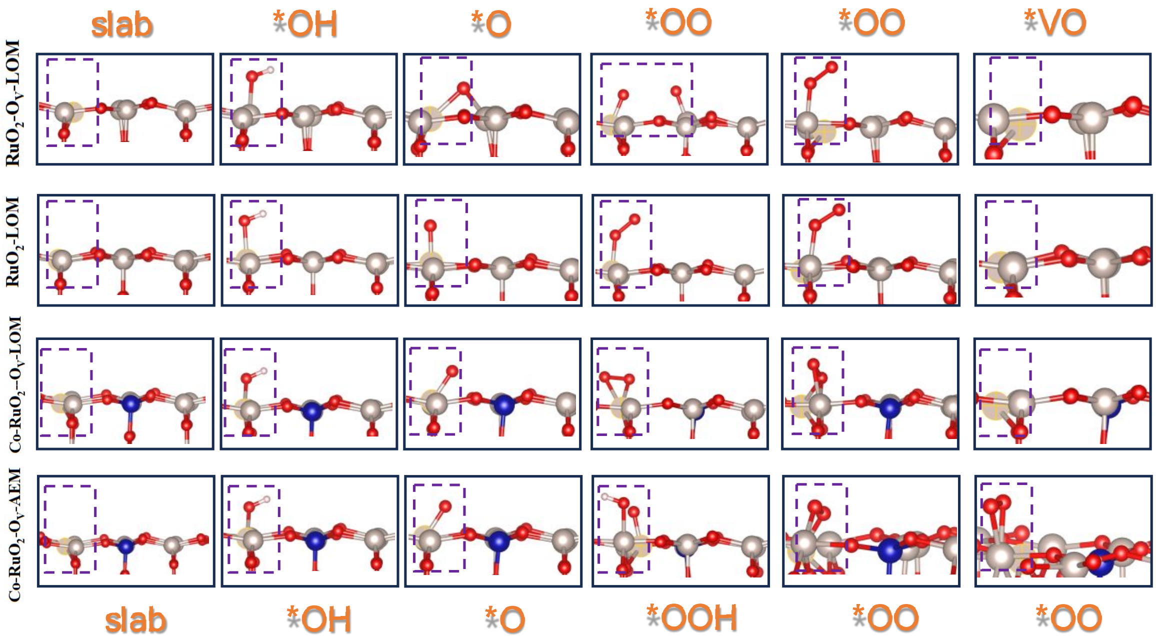

Figure S16. OER paths for RuO_2-LOM, RuO_2-O_V-LOM, Co-RuO_2-O_V-LOM, and Co-RuO_2-O_V-AEM.

Table S1. Co content of catalysts measured by ICP-AES/MS.

Catalysts	Co-element content (%)
RuO_2	0
$Co_{0.033}$-RuO_2	1.438%
$Co_{0.047}$-RuO_2	2.903%
$Co_{0.083}$-RuO_2	5.787%
$Co_{0.108}$-RuO_2	9.932%
$Co_{0.167}$-RuO_2	16.053%

Table S2. The O_V content of each catalyst calculated by O 1s spectroscopy.

Catalysts	O_V content (%)
RuO_2	28.9%
$Co_{0.033}$-RuO_2	29.6%
$Co_{0.047}$-RuO_2	30.7%
$Co_{0.083}$-RuO_2	31.4%
$Co_{0.108}$-RuO_2	33.7%
$Co_{0.167}$-RuO_2	36.3%

Table S3. Testing the actual volume of oxygen produced by $Co_{0.108}$-RuO_2 and $Co_{0.083}$-RuO_2 in an acidic electrolyte and the corresponding Faraday efficiency.

Electrolyte	Samples	Experimental O_2/ml	FE/%
0.5M H_2SO_4	$Co_{0.108}$-RuO_2	4.17	99.76
	$Co_{0.083}$-RuO_2	4.16	99.5

Table S4. Comparison of the surface parameters and mass activity for electrocatalysts investigated in 0.5 M H_2SO_4.

Sample	C_{dl} (mF cm^{-2})	R_f [a]	Surface area [b] (m^2 g_{oxide}^{-1})	Mass activity [c] (mA mg_{oxide})	ECSA [d] (mA cm^{-2})
RuO_2	21.4	356.7	125.2	52.2	356.7
$Co_{0.167}$-RuO_2	27.2	453.3	158.9	97.2	453.3
$Co_{0.108}$-RuO_2	33.5	558.3	195.8	101.9	558.3
$Co_{0.083}$-RuO_2	30.8	513.3	180.1	83.7	513.3
$Co_{0.047}$-RuO_2	29.2	486.7	170.7	97.4	486.7
$Co_{0.033}$-RuO_2	22.8	380	133.3	87.6	380
Com-RuO_2	7.85	130.8	45.9	18	130.8

a: R_f is calculated by dividing C_{dl} by the capacitance of an ideal planar metal oxide with a smooth surface (C_s is 0.06 mF cm^{-2}).

b: the surface area was calculated by multiplying the electrode geometrical area by R_f and then normalized by taking into account the loading mass of electrocatalysts;

c: the mass activity was obtained from the current density values at an overpotential of 270 mV.

d: ECSA is calculated as R_f multiplied by the geometric area of the catalyst.

Table S5. Comparison of the overpotentials of $Co_{0.108}$-RuO_2 with recently reported

Catalyst	Electrolyte	η at 10 mA cm^{-2} (mV)	Reference
**$Co_{0.108}$-RuO_2 NSs**	0.5M H_2SO_4	214	This work
**C-RuO_2**	0.5M H_2SO_4	318	This work
**Ru–N–C**	0.5M H_2SO_4	267	Ref.[1]
**High-entropy Ru alloys**	0.5M H_2SO_4	258	Ref.[2]
**RuCu nanosheets**	0.5M H_2SO_4	236	Ref.[3]
**RuO_2 nanowires**	0.5M H_2SO_4	234	Ref.[4]
**Amorphous $RuTe_2$**	0.5M H_2SO_4	245	Ref.[2, 5]
**$Y_{1.7}Sr_{0.3}Ru_2O_7$**	0.5M H_2SO_4	264	Ref.[6]
**Co-doped RuO_2 NWs**	0.5M H_2SO_4	230	Ref.[7]
**MnFeRu-90**	0.1 M $HClO_4$	270	Ref.[8]
**Bimetallic $Ir_{0.7}Ru_{0.3}O_2$**	0.05M H_2SO_4	225	Ref.[9]
**$Yb_2Ru_2O_7$**	0.1 M $HClO_4$	310	Ref.[10]
**Ru@IrO_x**	0.05M H_2SO_4	282	Ref.[11]
**$Ru_{0.9}(NiCo_{1.5})_{0.1}O_\delta$**	0.1 M $HClO_4$	280	Ref.[12]
**a-Pt/RuO2**	0.1 M $HClO_4$	227	Ref.[13]
**Si-RuO_2−0.1**	0.1 M $HClO_4$	226	Ref.[14]
**Ru_2Co_1Bo-350**	0.5M H_2SO_4	219	Ref.[15]
**Ru@Cr-FeMOF**		230	Ref.[16]
**$Y_2Ru_{1.2}Ir_{0.8}O_7$**	0.1 M $HClO_4$	220	Ref.[17]
**Co-Ru@RuO_x /NCN**	0.5M H_2SO_4	230	Ref.[18]

Table S6. Table of OER Gibbs free energy changes (ΔG) for RuO_2-LOM, RuO_2-O_V-LOM, Co-RuO_2-O_V-LOM, and Co-RuO_2-O_V-AEM

Catalysts	ΔG1 (eV)	ΔG2 (eV)	ΔG3 (eV)	ΔG4 (eV)	ΔG5 (eV)	ΔG6 (eV)	ΔG of rate-determining step (eV)
**RuO_2-LOM**	-0.515	1.003	3.428	2.224	-0.678	-0.543	**3.428**
**RuO_2-O_V-LOM**	-0.630	0.600	0.799	3.308	1.385	-0.542	**3.308**
**Co-RuO_2-O_V-LOM**	1.595	0.245	2.905	0.419	1.629	-1.874	**2.905**
**Co-RuO_2-O_V-AEM**	-0.541	0.072	0.142	2.599	0.466	2.181	**2.599**

References

(1) Cao, L.; Luo, Q.; Chen, J.; Wang, L.; Lin, Y.; Wang, H.; Liu, X.; Shen, X.; Zhang, W.; Liu, W.; et al. Dynamic Oxygen Adsorption on Single-atomic Ruthenium Catalyst with High Performance for Acidic Oxygen Evolution Reaction. *Nature Communications* **2019**, *10*, 4849 (2019), DOI: 10.1038/s41467-019-12886-z.

(2) Cai, Z.X.; Goou, H.; Ito, Y.; Tokunaga, T.; Miyauchi, M.; Abe, H.; Fujita, T. Nanoporous Ultra-high-entropy Alloys Containing Fourteen Elements for Water Splitting Electrocatalysis. *Chemical Science* **2021**, *12* (34), 11306-11315, DOI: 10.1039/d1sc01981c.

(3) Yao, Q.; Huang, B.; Zhang, N.; Sun, M.; Shao, Q.; Huang, X. Channel-Rich RuCu Nanosheets for pH-Universal Overall Water Splitting Electrocatalysis. *Angewandte Chemie-International Edition* **2019**, *58* (39), 13983-13988, DOI: 10.1002/anie.201908092.

(4) Yang, J.; Ji, Y.; Shao, Q.; Zhang, N.; Li, Y.; Huang, X. A Universal Strategy to Metal Wavy Nanowires for Efficient Electrochemical Water Splitting at pH-Universal Conditions. *Advanced Functional Materials* **2018**, *28* (41), 1803722, DOI: 10.1002/adfm.201803722.

(5) Wang, J.; Han, L.; Huang, B.; Shao, Q.; Xin, H. L.; Huang, X. Amorphization Activated Ruthenium-tellurium Nanorods for Efficient Water Splitting. *Nature Communications* **2019**, *10*, 5692 (2019) DOI: 10.1038/s41467-019-13519-1.

(6) Kim, J.; Shih, P.C.; Tsao, K.C.; Pan, Y.T.; Yin, X.; Sun, C.J.; Yang, H. High-Performance Pyrochlore-Type Yttrium Ruthenate Electrocatalyst for Oxygen Evolution Reaction in Acidic Media. *Journal of the American Chemical Society* **2017**, *139* (34), 12076-12083, DOI: 10.1021/jacs.7b06808.

(7) Wang, J.; Ji, Y.; Yin, R.; Li, Y.; Shao, Q.; Huang, X. Transition Metal-doped Ultrathin RuO_2 Networked Nanowires for Efficient Overall Water Splitting Across a Broad pH Range. *Journal of Materials Chemistry A* **2019**, *7* (11), 6411-6416, DOI: 10.1039/c9ta00598f.

(8) Wu, Y.; Tariq, M.; Zaman, W. Q.; Sun, W.; Zhou, Z.; Yang, J. Bimetallic Doped RuO_2 with Manganese and Iron as Electrocatalysts for Favorable Oxygen Evolution Reaction Performance. *Acs Omega* **2020**, *5* (13), 7342-7347, DOI: 10.1021/acsomega.9b04237.

(9) Saveleva, V. A.; Wang, L.; Luo, W.; Zafeiratos, S.; Ulhaq-Bouille, C.; Gago, A. S.; Friedrich, K. A.; Savinova, E. R. Uncovering the Stabilization Mechanism in Bimetallic Ruthenium-Iridium Anodes for Proton Exchange Membrane Electrolyzers. *Journal of Physical Chemistry Letters* **2016**, *7* (16), 3240-3245, DOI: 10.1021/acs.jpclett.6b01500.

(10) Liu, H.; Wang, Z.; Li, M.; Zhao, X.; Duan, X.; Wang, S.; Tan, G.; Kuang, Y.; Sun, X. Rare-earth-regulated Ru-O Interaction Within the Pyrochlore Ruthenate for Electrocatalytic Oxygen Evolution in Acidic Media. *Science China-Materials* **2021**, *64* (7), 1653-1661, DOI: 10.1007/s40843-020-1571-y.

(11) Shan, J.; Guo, C.; Zhu, Y.; Chen, S.; Song, L.; Jaroniec, M.; Zheng, Y.; Qiao, S.Z. Charge-Redistribution-Enhanced Nanocrystalline Ru@IrOx Electrocatalysts for Oxygen Evolution in Acidic Media. *Chem* **2019**, *5* (2), 445-459, DOI: 10.1016/j.chempr.2018.11.010.

(12) Wu, Y.; Tariq, M.; Zaman, W. Q.; Sun, W.; Zhou, Z.; Yang, J. Ni-Co Codoped RuO_2 with Outstanding Oxygen Evolution Reaction Performance. *Acs Applied Energy Materials* **2019**, *2* (6), 4105-4110, DOI: 10.1021/acsaem.9b00266.

(13) Hu, Y.; Han, X.; Hu, S.; Yu, G.; Chao, T.; Wu, G.; Qu, Y.; Chen, C.; Liu, P.; Zheng, X.; et al. Surface-Diffusion-Induced Amorphization of Pt Nanoparticles over Ru Oxide Boost Acidic Oxygen Evolution. *Nano Letters* **2024**, *24* (17), 5324-5331, DOI: 10.1021/acs.nanolett.4c01036.

(14) Ping, X.; Liu, Y.; Zheng, L.; Song, Y.; Guo, L.; Chen, S.; Wei, Z. Locking the Lattice Oxygen in RuO_2 to Stabilize Highly Active Ru Sites in Acidic Water Oxidation. *Nature Communications* **2024**, *15* (1), 2501 (2024), DOI: 10.1038/s41467-024-46815-6.

(15) Shen, L.W.; Wang, Y.; Chen, J.B.; Tian, G.; Xiong, K.Y.; Janiak, C.; Cahen, D.; Yang, X.Y. A RuCoBO Nanocomposite for Highly Efficient and Stable Electrocatalytic Seawater Splitting. *Nano Letters* **2023**, *23* (3), 1052-1060, DOI: 10.1021/acs.nanolett.2c04668.

(16) Zhao, C.; Wang, J.; Gao, Y.; Zhang, J.; Huang, C.; Shi, Q.; Mu, S.; Xiao, Q.; Huo, S.; Xia, Z.; et al. D-Orbital Manipulated Ru Nanoclusters for High-Efficiency Overall Water Splitting at Industrial-Level Current Densities. *Advanced Functional Materials* **2024**, *34* (7), 2307917, DOI: 10.1002/adfm.202307917.

(17) Liu, H.; Zhang, Z.; Li, M.; Wang, Z.; Zhang, X.; Li, T.; Li, Y.; Tian, S.; Kuang, Y.; Sun, X. Iridium Doped Pyrochlore Ruthenates for Efficient and Durable Electrocatalytic Oxygen Evolution in Acidic Media. *Small* **2022**, *18* (30), 2202513, DOI: 10.1002/smll.202202513.

(18) Wang, H.; Yang, P.; Sun, X.; Xiao, W.; Wang, X.; Tian, M.; Xu, G.; Li, Z.; Zhang, Y.; Liu, F.; et al. Co-Ru Alloy Nanoparticles Decorated onto Two-dimensional Nitrogen Doped Carbon Nanosheets towards Hydrogen/Oxygen Evolution Reaction and Oxygen Reduction Reaction. *Journal of Energy Chemistry* **2023**, *87*, 286-294, DOI: 10.1016/j.jechem.2023.08.039.
